# Glioma stem cells activate platelets by plasma-independent thrombin production to promote glioblastoma tumorigenesis

**DOI:** 10.1093/noajnl/vdac172

**Published:** 2022-11-07

**Authors:** Anthony R Sloan, Christine Lee-Poturalski, Harry C Hoffman, Peggy L Harris, Theresa E Elder, Brian Richardson, Amber Kerstetter-Fogle, Gino Cioffi, Julia Schroer, Ansh Desai, Mark Cameron, Jill Barnholtz-Sloan, Jeremy Rich, Eckhard Jankowsky, Anirban Sen Gupta, Andrew E Sloan

**Affiliations:** Department of Neurological Surgery, Case Western Reserve University, Cleveland, Ohio, USA; Department of Neurosciences, Case Western Reserve University, Cleveland, Ohio, USA; Department of Neurological Surgery, University Hospitals Cleveland Medical Center, Cleveland, Ohio, USA; Case Comprehensive Cancer Center, Case Western Reserve University, Cleveland, Ohio, USA; Case Comprehensive Cancer Center, Case Western Reserve University, Cleveland, Ohio, USA; Center for RNA Science and Therapeutics, Case Western Reserve University, Cleveland, Ohio, USA; Department of Neurological Surgery, Case Western Reserve University, Cleveland, Ohio, USA; Department of Neurological Surgery, Case Western Reserve University, Cleveland, Ohio, USA; Department of Neurological Surgery, University Hospitals Cleveland Medical Center, Cleveland, Ohio, USA; Case Comprehensive Cancer Center, Case Western Reserve University, Cleveland, Ohio, USA; Department of Neurological Surgery, Case Western Reserve University, Cleveland, Ohio, USA; Department of Neurological Surgery, University Hospitals Cleveland Medical Center, Cleveland, Ohio, USA; Case Comprehensive Cancer Center, Case Western Reserve University, Cleveland, Ohio, USA; Department of Population and Quantitative Health Science, Case Western Reserve University, Cleveland, Ohio, USA; Department of Neurological Surgery, Case Western Reserve University, Cleveland, Ohio, USA; Case Comprehensive Cancer Center, Case Western Reserve University, Cleveland, Ohio, USA; Division of Cancer Epidemiology and Genetics, Trans-Divisional Research Program, National Cancer Institute, Bethesda, Maryland, USA; Geisinger Commonwealth School of Medicine, Scranton, Pennsylvania, USA; Department of Neurological Surgery, Case Western Reserve University, Cleveland, Ohio, USA; Case Comprehensive Cancer Center, Case Western Reserve University, Cleveland, Ohio, USA; Department of Population and Quantitative Health Science, Case Western Reserve University, Cleveland, Ohio, USA; Division of Cancer Epidemiology and Genetics, Trans-Divisional Research Program, National Cancer Institute, Bethesda, Maryland, USA; Center for Biomedical Informatics and Information Technology, National Cancer Institute, Bethesda, Maryland, USA; Division of Hematology/Oncology, University of Pittsburgh School of Medicine, Pittsburgh, Pennsylvania, USA; Case Comprehensive Cancer Center, Case Western Reserve University, Cleveland, Ohio, USA; Center for RNA Science and Therapeutics, Case Western Reserve University, Cleveland, Ohio, USA; Case Comprehensive Cancer Center, Case Western Reserve University, Cleveland, Ohio, USA; Department of Biomedical Engineering, Case Western Reserve University School of Engineering, Cleveland, Ohio, USA; Department of Neurological Surgery, Case Western Reserve University, Cleveland, Ohio, USA; Department of Neurological Surgery, University Hospitals Cleveland Medical Center, Cleveland, Ohio, USA; Case Comprehensive Cancer Center, Case Western Reserve University, Cleveland, Ohio, USA; Department of Neurosciences, Piedmont Health, Atlanta Georgia, USA

**Keywords:** coagulation, glioma stem cells, thrombin

## Abstract

**Background:**

The interaction between platelets and cancer cells has been underexplored in solid tumor models that do not metastasize, for example, glioblastoma (GBM) where metastasis is rare. Histologically, it is known that glioma stem cells (GSCs) are found in perivascular and pseudsopalisading regions of GBM, which are also areas of platelet localization. High platelet counts have been associated with poor clinical outcomes in many cancers. While platelets are known to promote the progression of other tumors, mechanisms by which platelets influence GBM oncogenesis are unknown. Here, we aimed to understand how the bidirectional interaction between platelets and GSCs drives GBM oncogenesis.

**Methods:**

Male and female NSG mice were transplanted with GSC lines and treated with antiplatelet and anti-thrombin inhibitors. Immunofluorescence, qPCR, and Western blots were used to determine expression of coagulation mechanism in GBM tissue and subsequent GSC lines.

**Results:**

We show that GSCs activate platelets by endogenous production of all the factors of the intrinsic and extrinsic coagulation cascades in a plasma-independent manner. Therefore, GSCs produce thrombin resulting in platelet activation. We further demonstrate that the endogenous coagulation cascades of these cancer stem cells are tumorigenic: they activate platelets to promote stemness and proliferation *in vitro* and pharmacological inhibition delays tumor growth *in vivo*.

**Conclusions:**

Our findings uncover a specific preferential relationship between platelets and GSCs that drive GBM malignancies and identify a therapeutically targetable novel interaction.

**Key Points**

- GSCs endogenously produce all members of the extrinsic and intrinsic coagulation cascade, to produce thrombin to activate platelets and enhance GSC phenotypes.
- Pharmacological inhibition of the endogenous coagulation cascade of GSCs slows tumor growth *in vivo*.

**Importance of the Study**

A hypercoagulable and hyper-thrombotic state are well-established phenomena in patients with GBM and many other malignancies. Here we show that subpopulations of glioma cells contribute to this hyperthrombotic state intra-tumorally. We demonstrate that GSCs co-opt and activate platelets to promote GBM tumor progression. GSCs endogenously produce all coagulation factors of the intrinsic and extrinsic cascade generating thrombin and activating platelets in the absence of plasma. Conversely, inhibition of platelet activation and thrombin production by GSCs abrogates platelet-mediated GSC self-renewal and growth. Similarly, inhibiting intratumoral thrombin production and function decreases tumor formation *in vivo.* These studies demonstrate that cancer stem cells readily execute a highly liver-specific gene expression program that is mechanistically linked to GBM tumor progression.

The tumor microenvironment (TME) in glioblastoma (GBM) is characterized by regions of necrosis, neoangiogenesis, and invasion.^1–3^ Glioma stem cells (GSCs) comprise a small subpopulation of the TME in GBM and are self-renewing, multipotent, and believed to be responsible for treatment resistance and progression.^1–3^ Platelets, anucleate blood cells, which primarily function in hemostasis, thrombosis, and wound healing are thought to be a critical contributor to disease progression in metastatic malignancies,^1–5^ but their importance in GBM, a disease that rarely metastasizes, has not previously been described. Similarly, there is little understanding of the cellular interactions between GSCs and platelets and their potential functional interaction in the TME.

Intratumoral thrombosis and necrosis are a hallmarks of GBM and systemic thrombocytosis is a negative prognosticator, but the etiology and functional importance of this phenomenon with respect to oncogenesis are poorly understood.^1–3,6,7^ During the initiation of thrombosis and coagulation, platelets become activated and release several inflammatory mediators and growth factors, resulting in interactions with other cell types, such as neutrophils, monocytes, and lymphocytes.^8^ Conversely, in their inactive state, platelets do not interact with other cell types, suggesting that platelet activation is necessary for their function.

Thrombin is one of the major drivers of platelet activation. Thrombin production in canonical coagulation mechanisms depends on coagulation factors that, to date, are primarily produced and secreted into plasma by hepatocytes. Previous work has suggested the production of a small subset of coagulation factors that make up the extrinsic coagulation cascade (tissue factor and FVll), are produced by other cell types,^9–13^ however, a complete functional coagulation mechanism has not been reported outside of hepatocytes.

Here we demonstrate that GSCs produce all coagulation factors necessary to generate thrombin endogenously without exposure to plasma; resulting in platelet activation and enhancing GSC proliferation and self-renewal. Similarly, pharmacological inhibition of factor X (FX) and thrombin at physiological doses decreases GBM oncogenesis *in vivo* consistent with these findings. The demonstration that GSCs produce all the components of the coagulation cascade is novel, as is the demonstration that inhibition of these components decreases oncogenesis *in vitro* and *in vivo.* This finding also serves as an exemplar of how cancer cells (like GSCs) can co-opt non-cancer cells to promote their own growth. This finding demonstrates how GSCs contribute to the hyper-thrombotic TME. which is known to play a role in GBM oncogenesis and identifies novel potential therapeutic targets by targeting GSC-specific coagulation rather than systemic coagulation.

## Materials and Methods

Detailed methodological descriptions and antibodies are available in Supplementary material.

### Patient Samples

All human patient tumor tissue and blood samples were acquired from newly diagnosed or recurrent GBM patients 18 and older seen for clinical care at University Hospitals-Seidman Cancer Center in compliance with protocols approved by the University Hospitals Institutional Review Board (IRB) and following informed consent.

### Immunofluorescence Staining

GBM tissues were fixed in 4% paraformaldehyde, followed by incubations in graded sucrose prior to embedding in the OCT compound. Samples were sectioned at 20 µm. Antibodies in supplementary methods.

### GBM-Platelet Survival Analysis

Kaplan–Meier analyses were performed and median survival in months, with corresponding 95% confidence intervals, are reported. Univariate and multivariable Cox proportional hazards were performed to assess the impact of thrombocyte count on overall survival, and hazard ratios are reported.

### Western Blot Analysis

Thirty micrograms of protein per sample were loaded in Laemmli sample buffer containing 2-mercaptoethanol onto a precast Mini-Protean TGX gel, 4–10%, and separated at 100 V before transfer to a PDVF membrane via the wet transfer method at 80 V for 2 h. Antibodies used are in Supplementary methods.

### Quantitative Real Time PCR (qPCR)

Gene expression was measured using the SYBR Green Supermix protocol and BIO-RAD CFX Connect Real Time System. Please refer to Supplementary Table S4 for PCR primer sequences.

### Human Thrombin ELISA

GSC thrombin secretion was measured using a human thrombin simplestep ELISA^®^ Kit and the subsequent protocol.

### Cell Titer Glo

Proliferation assays using the CellTiter Glo^®^ Luminescent Cell Viability Assay were conducted following manufacturing protocol.

### Colony Formation Assay

GSC lines were incubated with platelets from either healthy subjects or tumor patients for a colony formation assay to measure sphere formation.

### Platelet Aggregation Assay

PRP was loaded into a microcuvette and incubated for 5 min at 37°C. Platelet aggregation was initiated by adding 50 µL of 2 units/mL human thrombin or conditioned media.

### Flow Cytometry Assay to Measure Platelet Activation

Platelets were isolated as described in supplemental methods. Antibodies used to measure platelet activation are in the supplementary methods.

### In vivo Flank Antithrombin Experiments

GSC3691 cells were suspended in HBSS and injected at a ratio of 1:1 with geltrex. Over the next 6–7 days tumors developed to the point where a small mass was visual. Upon tumor formation, animals received daily IP injections of treatment. Detailed methodology in supplementary methods.

### Orthotopic Xenograft Model

Intracranial xenografting was completed as previously described.^14^ U87 GBM cells were intracranially injected, upon tumor formation and treatments started. Mice were treated for 21 days with IP injections of either 30 mg/kg clopidogrel (*n* = 8), 10 mg/kg apixaban (*n* = 8), 30 mg/kg dabigatran (*n* = 8), or vehicle control. All animal experiments were in accordance with Case Western Reserve University IACUC policies.

### Data Availability

Data supporting the findings of this study are available within the paper and its Supplementary Information. Source data are provided in this paper.

## Results

### Glioblastoma Stem Cells and Platelets Localization in GBM

**Figure 1.**
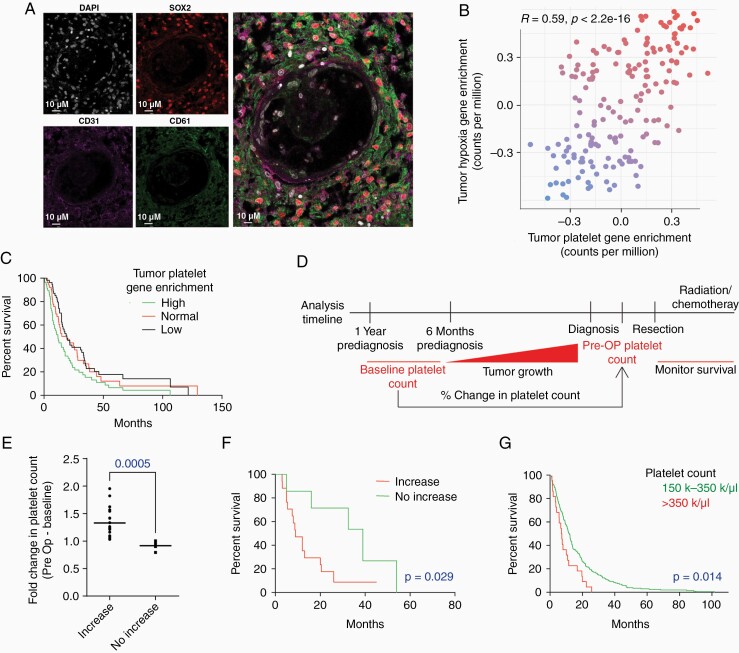
Correlation between platelets, GSCs, and GBM patient outcomes. (a) Immunofluorescence (IF) staining of platelets and GSCs in GBM patient tissues; IF staining of DAPI, SOX2, CD31, and CD61. Merged image of DAPI, SOX2, CD31, and CD61 IF staining. (scale = 10µm). (b) TCGA analysis comparing hypoxia enrichment and platelet enrichment in GBM patient tissue. Points are colored by the first principal component. (c) Kaplan–Meier survival analysis of the TCGA-GBM cohort stratified into tertiles by enrichment levels of the Raghavachari platelet gene signature. (d) Experimental timeline for the analysis of our first patient cohort at University Hospital-Seidman Cancer Center assessing the correlation of the percent change in the platelet count with prior to diagnosis to survival. (e) Median 31% increase in platelet count between patients (*n* = 20) who had an increase in the platelet count from baseline (>6 months prior to diagnosis) to just prior to surgery and those who had no increase (*n* = 204). (f) Kaplan–Meier survival analysis comparing GBM patients with an increase vs. no increase in platelet counts prior to diagnosis. (g) Kaplan–Meier survival analysis from a second cohort of patients at University Hospital-Seidman Cancer Center demonstrating that GBM patients with high platelet counts (*n* = 20; >350,000 k/µL) had shorter survivals than those with normal platelet counts (*n* = 224; 150–350 k/µL).

Platelets have been localized to well-established GSC-related niches, specifically necrotic and hypoxic regions of the tumor, in prior studies, but expression proximal to GSC has not been described.^6,7,15–17^ Immunofluorescence (IF) staining of GBM patient specimens demonstrated SOX2-positive tumor cells (GSCs marker) and CD61-positive platelets in and around the vasculature, identified by CD31 (Figure 1a, Supplementary Figure S1). These results indicate that GSCs and platelets localize proximal to one another in the GBM TME.

### Correlation Between Platelets and GSC Gene Signatures in GBM

Thrombocytosis is a negative prognostic factor in GBM but the mechanism is unknown.^1–3,6,7,15^ To distinguish the functional importance of high platelet counts on overall GBM oncogenesis, we sought to determine if a correlation was present between platelets and GSCs in GBM specimens. Interrogating the National Institute of Health’s Cancer Genome Atlas Glioblastoma Multiforme (TCGA-GBM) database revealed a strong correlation between tumor platelet gene and tumor hypoxia gene signatures (indirect GSC signature) in GBM (Figure 1b).^18,19^ In addition, analyses of the TCGA patient cohort demonstrated that GBM patients with the highest gene expression of the platelet-related gene signature exhibited significantly shorter survival times compared to GBM patients with lower expression (Figure 1c), further indicating a correlative relationship between platelets and GSCs in GBM.^19^

Similarly, both platelet and GSC gene signatures were preferentially expressed in high-grade (grade IV, GBM) tissue specimens compared to lower-grade gliomas (Supplementary Figure S2a–d). Clinical implications of high platelet counts in GBM were substantiated by comparing cohorts of patients with “high platelet” counts (>350 platelets/100 000 cells) and “normal platelet” counts (150–300 platelets/100 000 cells) at University Hospital-Seidman Cancer Center. Though there was no statistical difference in the demographics of the patients in the two groups (Supplementary Table S1) elevated platelet counts correlated with poor overall and median survival in GBM patients (Figure 1d–g, Supplementary Table S2–S3).

### Platelets Promote the Growth and Stemness Phenotypes of GSCs

**Figure 2.**
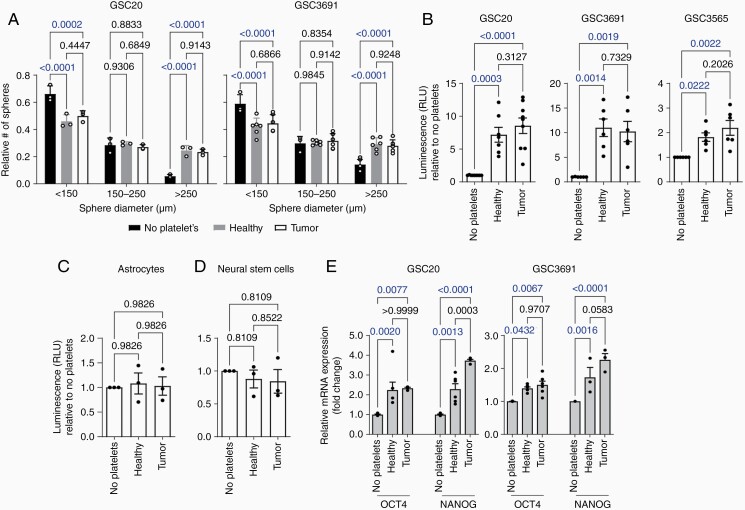
Platelet promotes the growth and stemness phenotypes of GSCs. (a) Measurement of the self-renewal capacity of GSC20 and GSC3691 upon exposure to platelets (1 GSC: 10 platelets) from healthy subjects or GBM patients as analyzed by determining the normalized cumulative distribution of sphere diameters 10 days after platelet exposure. (b) GSC proliferation upon exposure to platelets from healthy subjects or GBM patients (1 GSC: 10 platelets) normalized to GSC growth in the absence of platelets (“no platelets”) for 3 different patient-derived GSC lines (Mes20 GSC, GSC3691, and GSC3565). (c) Proliferation of non-malignant human astrocytes upon exposure to platelets (1 astrocyte: 10 platelets) from healthy subjects or GBM patients normalized to astrocyte proliferation upon exposure to no platelets. (d) Proliferation of neural stem cells exposed to platelets (1 neural stem cell: 10 platelets) from healthy subjects or GBM patients normalized to neural stem cell proliferation upon exposure to no platelets. (e) Quantitative PCR analysis of the transcriptional expression of OCT4 and NANOG in GSC20 and GSC3691 upon exposure to platelets from healthy subjects or tumor patients (1 GSC: 10 platelets). All data are presented as the mean ± SEM circles representing separate experiments. Comparisons were made using two-way independent *t*-test and one-way ANOVA. Qualitative data, including IF microscopy are representative images from multiple different experiments.

Given platelets and GSC localization in the same niches of the tumor microenvironment in GBM, we tested whether platelets promote GSC growth. Platelets from GBM patients and healthy subjects were isolated and incubated with GSCs under serum-free conditions. Platelets derived from either healthy subjects or GBM patients lead to increases in GSC sphere size (Figure 2a, Supplementary Figure S3a and b) as well as a 5 to 10-fold increase in proliferation (Figure 2b). In contrast, platelet exposure did not enhance the proliferation of non-transformed astrocytes (Figure 2c), neural stem cells (Figure 2d), or differentiated glioma cells (DGCs) (Supplementary Figure S3c and d). Further, platelets induced elevation of GSC stemness markers (OCT 4 and NANOG), as measured by mRNA levels (Figure 2e). These results demonstrate that platelets preferentially promote the proliferation and stemness of GSCs.

### Glioma Stem Cells Activate Platelets

**Figure 3.**
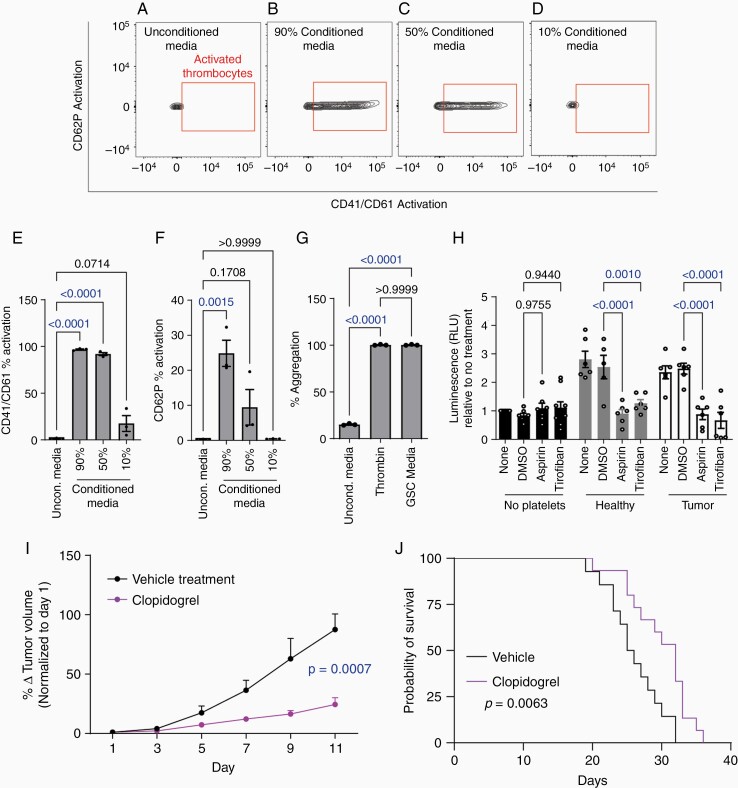
GSCs activate Platelets to promote tumorigenesis. (a–d) Flow cytometry analysis measuring platelet activation upon exposure to GSC3656-conditioned medium. Flow cytometry plots showing platelet activation (a) upon exposure to standard unconditioned medium, (b) upon exposure to 90% conditioned medium, (c) upon exposure to 50% conditioned medium, and (d) upon exposure to 10% conditioned medium. (e) Quantification of percent platelet activation, via CD41/CD61 expression, normalized to baseline activation. (f) Quantification of the percent platelet activation based on CD62P expression, normalized to baseline activation. (g) Aggregometry analysis showing platelet aggregation in response to 50% GSC3565-conditioned medium compared to unconditioned medium and thrombin. (h) Patient-derived GSC356 proliferation upon exposure to platelets (1 GSC: 10 platelets) from healthy or GBM patients relative to GSC exposed to no platelets with or without treatment with platelet activation inhibitors, aspirin, and tirofiban. (i) Inhibiting platelet activation with clopidogrel decreases GSC3691 tumor formation in a flank xenograft model. (j) Increases survival in an orthotopic xenograft model (U87); combined analysis from 2 separate independent experiments [Experiment 1: Vehicle (*n* = 6), Clopidogrel (*n* = 7); Experiment 2: Vehicle (*n* = 8). Clopdidogrel (*n* = 8)]. All data are presented as the mean ± SEM from three independent experiments.

Given that platelets enhance GSC growth and activated platelets interact with other cell types, we investigated whether GSCs themselves enhance platelet activation. Incubation with GSC-conditioned medium caused concomitant increases in CD41/CD61 active form and CD62P (platelet activation markers) expression in a dose-dependent manner (Figure 3a–d, quantified with normalization to baseline in Figure 3e–f), indicative of platelet activation. Platelet activation was further confirmed by an aggregometry assay using washed platelets, which demonstrated that GSC-conditioned medium caused platelet aggregation equivalent to the response to thrombin, one of the main platelet activators (Figure 3g).^20^ These results demonstrate that GSCs secrete a platelet activating factor.

### Platelet Activation is Required to Promote Platelet-Induced GSC Growth

We next determined whether platelet activation is required to enhance GSC growth and stemness phenotypes. We treated GSC and platelets with aspirin and tirofiban, both of which inhibit platelet activation.^17^ Both agents blocked platelet-induced proliferation of GSCs (Figure 3h). To determine whether platelet activation is required to promote GBM tumorigenesis, we used clopidogrel, to inhibit platelet aggregation and subsequent activation in an *in vivo* flank model and an orthotopic model. We found that clopidogrel inhibited tumor formation over time relative to vehicle control mice in our flank model (Figure 3i) and prolonged survival in our orthotopic model (Figure 3j, Supplementary Figure S4). This indicates that platelet activation is required for platelet stimulation of GSC tumorigenesis.

### GSCs Produce Thrombin

**Figure 4.**
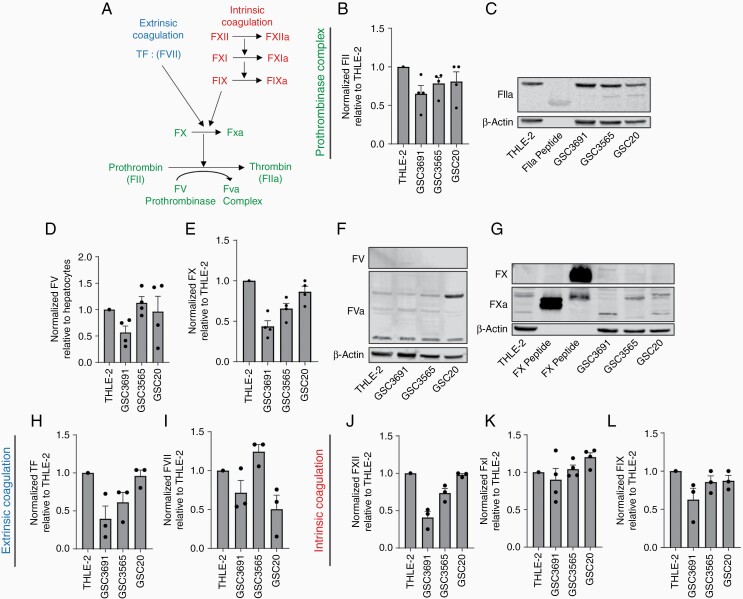
GSCs produce thrombin via the coagulation cascades. (a) Summary of the extrinsic and intrinsic coagulation pathways that are present in GSCs and are responsible for the endogenous production of Flla by Fll cleavage. (b, c) protein expression of Flla by GSC relative to hepatocytes (THLE-2) (b) Western blot quantification of the expression of FII normalized to hepatocytes (c) Western blotting for Flla expressed by GSC and hepatocytes relative to β-actin as a loading control. (d–g) Expression of coagulation factors that form the prothrombinase complex with Flla. (d) Western blot quantification of FV expression normalized to hepatocytes. (e) Western blot quantification of FX expression normalized to hepatocytes. Western blotting showing (f) FV/FVA and (g) FX/FXa expression relative to β-actin. (h, i) Expression of extrinsic coagulation factors in GSCs. Western blot quantification of (h) TF and (i) FVll. j–k, Expression of intrinsic coagulation factors in GSCs. Western blot quantification of (k) FXll, (k) FXl, and (l) FIX. All data are presented as the mean ± SEM of three independent experiments. THLE-2 = human adult hepatocytes.

We next sought to determine which platelet activating factor GSCs secrete in order to activate platelets (Figure 3). Thrombin is one of the main platelet activators and is the result of the coagulation cascade. The canonical coagulation cascade is comprised of two pathways: the intrinsic and extrinsic coagulation cascades, both of which involve a series of cleaved serine proteases that lead to platelet activation by thrombin. The coagulation factors are produced by hepatocytes and the coagulation cascades occur in the blood plasma.^21^ Both coagulation cascades ultimately lead to the cleavage of prothrombin (FII) to thrombin (Flla) (Figure 4a). Thrombin (FIIa) binds to platelets, resulting in platelet activation as well as the conversion of fibrinogen to fibrin, ultimately leading to clot formation.^22^

Cancer cells have thus far only been shown to generate thrombin production in the presence of plasma via expression of tissue factor, which interacts with factor Vll.^1,23–26^ However, we observed platelet activation by GSC-conditioned media, without any plasma (Figure 3). To examine the basis for this plasma-independent platelet activation, we assessed whether GSCs secreted thrombin to activate platelets. We indeed detected thrombin (Flla) secretion by GSCs of both proneural (GSC3691 and GSC3565) and mesenchymal (GSC20) subtypes (Supplementary Figure S5a).

To investigate whether Flla can be produced endogenously from Fll within GSCs, we measured the expression of Fll and FIIa mRNA and protein. GSCs expressed higher Fll mRNA expression than hepatocytes (Supplementary Figure S5b), and expressed both Fll and Flla at the protein level (Figure 4b–c, Supplementary Figure S6). We also detected expression of FV and FX at the mRNA (Supplementary Figure S5c–d) and protein levels (Figure 4d–g, Supplementary Figure S7). In addition, FV and FX were expressed in their respective cleaved (ie, activated) forms, FXa and FVa, in GSCs (Figure 4f and g). These results indicate that GSC endogenously produces all the coagulation factors of the prothrombinase complex in its respective active state.

### GSC Produce all the Coagulation Factors of the Extrinsic and Intrinsic Cascades

We next investigated whether GSCs produce the coagulation factors of the extrinsic, and or intrinsic cascades. GSCs endogenously expressed the coagulation factors of the extrinsic coagulation cascade including tissue factor (TF) and factor Vll (FVll) at both the mRNA (Supplementary Figure S8a–b) and protein levels (Figure 4h–I, Supplementary Figure S9, Supplementary Figure S10). FVll was present in both its precursor form (FVII) and in its active, cleaved form (FVlla) (Supplementary Figure S10b).

GSCs also co-expressed FXll, FXl, and FlX, which comprise the entire intrinsic coagulation cascade, at the mRNA (Supplementary Figure S8c–e, respectfully) and protein levels (Figure 4j–l, Supplementary Figure S11, and Supplementary Figure S12, respectfully). FXll, FXl, and FlX were present in their active cleaved forms (Supplementary Figure S12a–c). Although GSCs are responsible for the treatment-resistant seen in GBM, we also confirmed expressed of all these coagulation factors in the differentiated form of these GSCs (DGCs) (Supplementary Figure S13). Collectively, these results show that GSCs inherently express the coagulation factors of both the intrinsic and extrinsic coagulation pathways, as well as the prothrombinase complex. The presence of these cascades serves as the basis for the unprecedented plasma-independent Flla generation and secretion that occurs in GSCs and provides the framework for a mechanism of GSC-induced platelet activation (Figure 4a).

### Inhibiting FX Cleavage Decreases Endogenous Thrombin Production in GSCs

**Figure 5.**
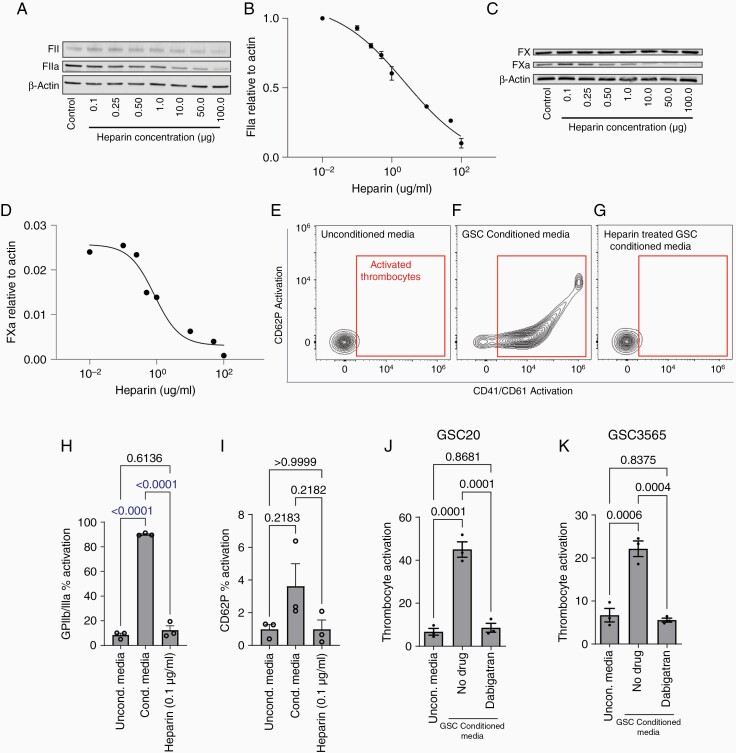
GSCs produce thrombin via a plasma-independent mechanism. (a, b) GSC3565 Fll cleavage into Flla upon heparin treatment in a dose-dependent manner measured by (a) Western blotting relative to β-actin as a loading control; (b) dose–response quantification of Flla Western blot. (c, d) GSC3565 FX cleavage into FXa upon heparin treatment in a dose-dependent manner measured by (c) Western blot relative to β-actin as a loading control; (d) quantification of Flla Western blot. (e–i) Flow cytometry plots for platelet activation upon exposure to conditioned medium from GSC3565 treated with heparin. (e) Baseline platelet activation; platelet activation (f), upon exposure to 90% conditioned medium; and (g), upon exposure to 90% conditioned medium from heparin-treated GSCs. (h) Quantification of percent platelet activation via CD41/CD61. (i) Quantification of the percent platelet activation relative to CD62P expression. (j, k) Quantification of flow cytometry measurements of percent platelet activation upon exposure to 60% conditioned media with and without the thrombin inhibitor, dabigatran in GSC20 and GSC3565, respectively.

To validate endogenous thrombin production by GSCs, we treated GSCs with low-molecular-weight heparin (LMWH) to prevent cleavage and subsequent activation of FXa. We expected a decrease in Fll conversion to Flla if thrombin was actively produced. The treatment of GSCs with LMWH resulted in concomitant decreases in Flla (Figure 5a and b) and FXa heavy chain (ie, active form) expression (Figure 5c and d) in a dose-dependent manner while the precursor levels of Fll and FX were unchanged. These data validate endogenous Flla production by GSCs.

### Inhibiting FX Cleavage in GSCs Decreases Platelet Activation by GSCs

**Figure 6.**
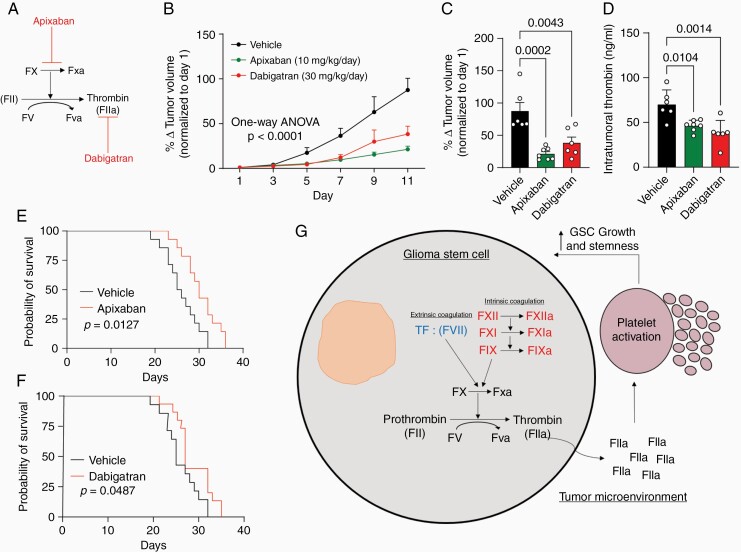
Pharmacological inhibition of FX and thrombin decreases oncogenesis in vivo. (a) Summary of the pharmacological inhibition of FX cleavage and thrombin activity using apixaban and dabigatran. (b, c) Inhibiting FX cleavage into FXa and inhibiting thrombin activation results in a decrease in GSC3691 tumor formation. (c) Tumor size at the final day (day 11) of treatment. (d) Inhibition of FX cleavage and thrombin activity decrease intratumoral thrombin expression *in vivo*. (e) Treatment with apixaban to inhibit FX cleavage significantly increases mouse survival in an orthotopic xenograft model (U87 Model). (f) Treatment with dabigatran to inhibit thrombin activity increases mouse survival in an orthotopic xenograft model (U87 Model). (g) Summary of the findings that GSCs hijack the coagulation cascade, to produce thrombin subsequently activating platelets, to enhance their growth and stemness. All data are presented as the mean ± SEM of 3 independent. Experiments analyzed using an unpaired *t*-test, one-way ANOVA, or log-rank test.

We next examined whether endogenous thrombin production is critical for platelet activation by GSCs. We determined whether inhibition of Flla production decreased platelet activation by GSCs. Exposure of platelets to GSC-conditioned medium derived from LMWH-treated GSCs resulted in significantly reduced CD41/CD61 and CD62P activation compared to untreated GSC-conditioned media (Figure 5e–i). This is due to the diminished production and secretion of thrombin by GSCs when treated with LMWH (Figure 5a–c). Similarly, adding dabigatran, a known thrombin inhibitor, to the co-culture of GSC-conditioned media and platelets inhibited platelet activation by GSC-conditioned media (Figure 5j–k). These results validate the finding that GSCs hijack the coagulation cascade to actively produce thrombin, which in turn activates platelets to promote GSC phenotypes (Figure 6g).

### Expression of Thrombin in GSC and Platelet Containing Niches in GBM Patient Tissue

Due to the finding that GSCs produce and secrete thrombin, we next determined whether thrombin was expressed in GBM patient tissue in areas that contain platelets and GSCs. IF staining of GBM patient specimens demonstrated Flla expression in CD61-positive platelets and SOX2-positive areas of the TME (Supplementary Figure S14). These results suggest thrombin expression throughout the tumor microenvironment in GBM patient specimens in areas containing GSCs and platelets.

### Pharmacological Inhibition of FX and Thrombin Slows Tumor Growth in vivo

To determine whether the plasma-independent thrombin production by GSC that we have identified is critical for tumorigenesis, we used apixaban, to inhibit factor X activation, and dabigatran to block thrombin’s active site in an *in vivo* flank model (Figure 6a). We found that apixaban and dabigatran both inhibited tumor formation over time relative to vehicle control mice (Figure 6b and c). Further, validation showed apixaban and dabigatran administration decreased intratumoral thrombin expression (Figure 6d). In the orthotopic model apixaban administration (Figure 6e) and dabigatran (Figure 6f) significantly prolonged mouse survival. These findings are consistent with a mechanistic link between plasma-independent thrombin production in GSCs, platelet activation by GSCs, and GBM oncogenesis (Figure 6g).

## Discussion

This work demonstrates 2 novel and important findings; first, cancer stem cells activate platelets without blood plasma by endogenously producing all the factors of the intrinsic and extrinsic coagulation cascades as well as the prothrombinase complex. This results in thrombin production and downstream platelet activation. Second, we demonstrate that the endogenous coagulation cascades in GSCs are oncogenic; they promote stemness and proliferation *in vitro* and pharmacological inhibition slows tumorigenesis and improves survival in an *in vivo* model. These findings demonstrate that GSCs co-opt the coagulation pathways to activate platelets in a plasma-free milieu. Although we acknowledge the role that a hyper-coagulative state has in cancer progression,^5,7,9,13,27,28^ this is the first time that the complete coagulation cascades is present, to actively produce thrombin, in any cell type outside of the liver.

Importantly, these *in vitro* and preclinical findings correlate with clinical prognosis in patients with GBM. We demonstrate that GBM patients who have an increase in platelet count prior to diagnosis have poorer survival times GBM patients. Although we acknowledge certain confound in contrast to patients who do not have an increase in their baseline platelet count. Similarly, a high preoperative platelet count at the time of diagnosis, as well as high levels of platelet gene signatures correlate with shorter survival time in GBM patients. Although we acknowledge certain confounding variables were not taken into consideration for these analyses, these clinical studies are consistent with previously reported findings.^23,24^

Equally important, platelet gene signature enrichment correlates with glioma stem cell enrichment. The correlation between platelets and stem cell transcriptional enrichment suggests cellular enrichment between these two cell types that is evident in GBM solid tissue (Figure 1a). Similarly, thrombin expression was seen throughout the GBM tumor microenvironment. Finally, we showed for the first time that exposing GSCs to platelets from both healthy or GBM patients enhanced the proliferation and stemness of the GSCs in multiple patient-derived GSC lines yet had no effect on the proliferation of neural stem cells, human astrocytes, and differentiated glioma cells (DGCs). Although some studies have shown the effect that tumor cells have on platelet activation,^29^ to our knowledge there have been no previous molecular studies demonstrating the effect of GSC on platelet activation. These studies show that GSC-conditioned media results in platelet activation (Figure 3) and inhibiting this platelet activation reverses the effects *in vitro* and *in vivo* leading us to further explore what factors GSCs are secreting to result in platelet activation.

The importance of thrombin in cancer pathology, and more specifically in glioma, has been previously described.^30–34^ Studies have demonstrated that thrombin induces glioma cell proliferation *in vitro*,^30^ and that treatment with Argatroban, a thrombin inhibitor, reduced glioma growth and prolonged survival on an *in vivo* model,^30,35^ suggesting that thrombin may play a role in glioma biology. However, while some coagulation factors are expressed by specific cancer cells, no cell type has shown the expression of the coagulation cascade entirely.^9,11,12,27,36^

In contrast, this study demonstrates that GSCs express the entire intrinsic and extrinsic cascades along with the prothrombinase complex resulting in thrombin production and secretion. Furthermore, we demonstrate a mechanistic link between the expression and secretion of these coagulation factors and GBM oncogenesis by which thrombocyte activation promotes stemness and proliferation of GSCs, suggesting a crucial GSC-platelets positive feedback loop that, when disrupted, may prove to be an effective therapeutic target in GBM (Figure 6g). This raises the possibility that selective local disruption of thrombin production in GSCs, in the TME inhibits tumor growth *in vivo* without systemic hyper-coagulation.

Sporadic expression of individual coagulation factors has been previously identified in both non-hepatic, malignant, and nonmalignant cell types. For example, FX and FV are expressed in endometrial cancer, breast cancer-derived immune cells, and in TME macrophages, endothelium, fibroblasts, and retinal pigment epithelial cells.^37–42^ TF and FVll have also been shown to be expressed in malignant cells such as ovarian, hepatocellular carcinoma, and GBM.^9,11,12,43,44^ However, previous studies have not demonstrated the expression and physiological function of *either* the entire extrinsic, the entire intrinsic coagulation factors or the entire prothrombinase complex components.

We thus propose that a novel bidirectional interaction takes place between platelets and GCSs whereby platelets exert oncogenic effects on GSCs by enhancing the self-renewal capacity and GSCs produce and secrete thrombin to enhance platelet activation, acting as a positive feedback loop for the tumorigenic effects of platelets on GSCs. Given the demonstrated link between thrombin and progression of several other tumor types, it is possible that other cancer cells also endogenously produce thrombin. Investigation of thrombin production in other tumor types could lead to a broader understanding of this specialized and cell type-specific crosstalk between platelets and cancer cells as modeled in GSCs.^30–34^ Our finding that the entire coagulation cascade is present and functional in non-hepatic cell types to produce, secrete and activate thrombin without plasma, requires a reassessment of how we understand and investigate pathophysiology of thrombosis in malignancies and other diseases. Similarly, our *in vivo* findings demonstrate how targeting this new phenomenon may be therapeutically relevant. Future studies will examine endogenous thrombin production, secretion, and activation in other cell types to determine the prevalence of this phenomenon and its therapeutic relevance.

## Supplementary Material

vdac172_suppl_Supplementary_Data_S1

vdac172_suppl_Supplementary_Data_S2

vdac172_suppl_Supplementary_Data_S3
