# Supplementary material for: Glioma stem cells activate platelets by plasma-independent thrombin production to promote glioblastoma tumorigenesis
Source: Neurooncol Adv. 2022 Nov 7;4(1):vdac172. doi: 10.1093/noajnl/vdac172 (PMC9700385; doi:10.1093/noajnl/vdac172)
Supplement: vdac172_suppl_Supplementary_Data_S1 [file vdac172_suppl_supplementary_data_s1.docx]

**Supplementary data:**

**
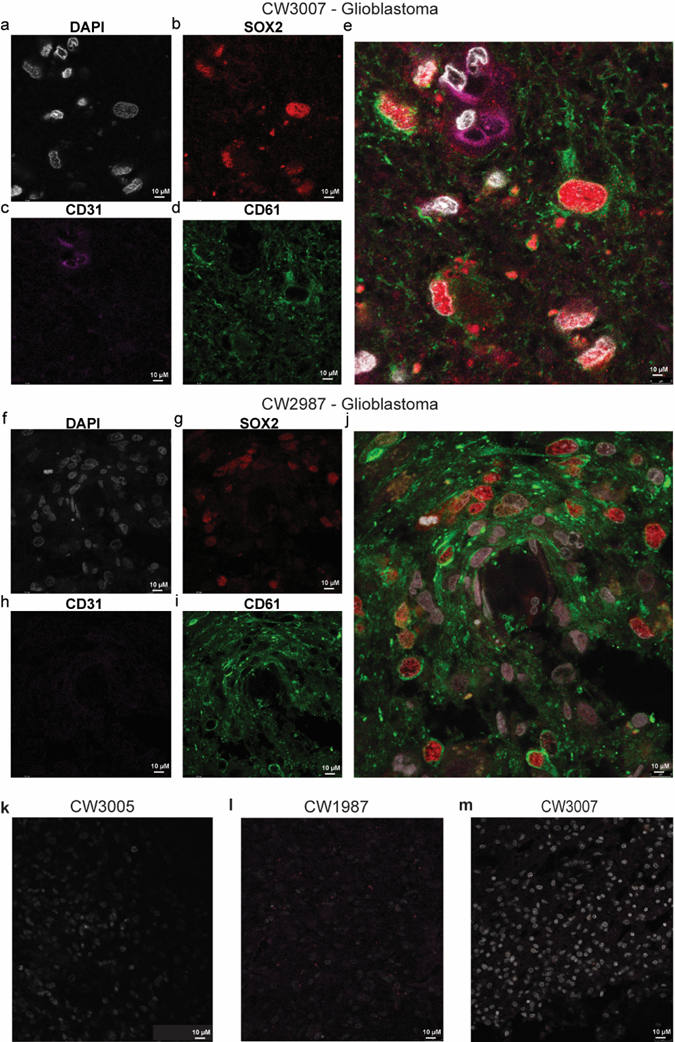
**

**Supplementary Fig. 1: Platelet and GSC expression in GBM patient tissue.**

**
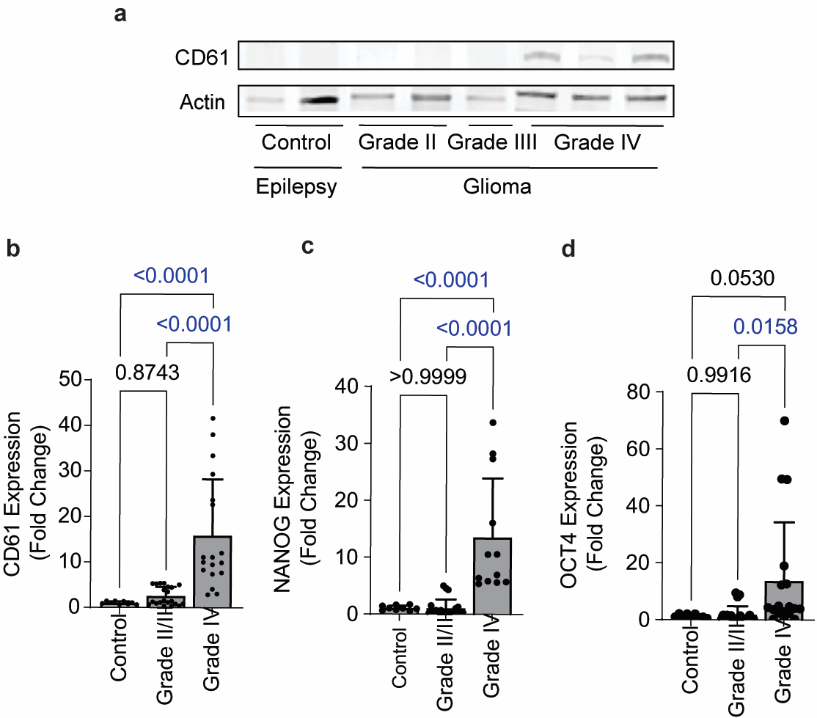
**

**Supplementary Fig. 2: CD61expression is specific to GBM.**


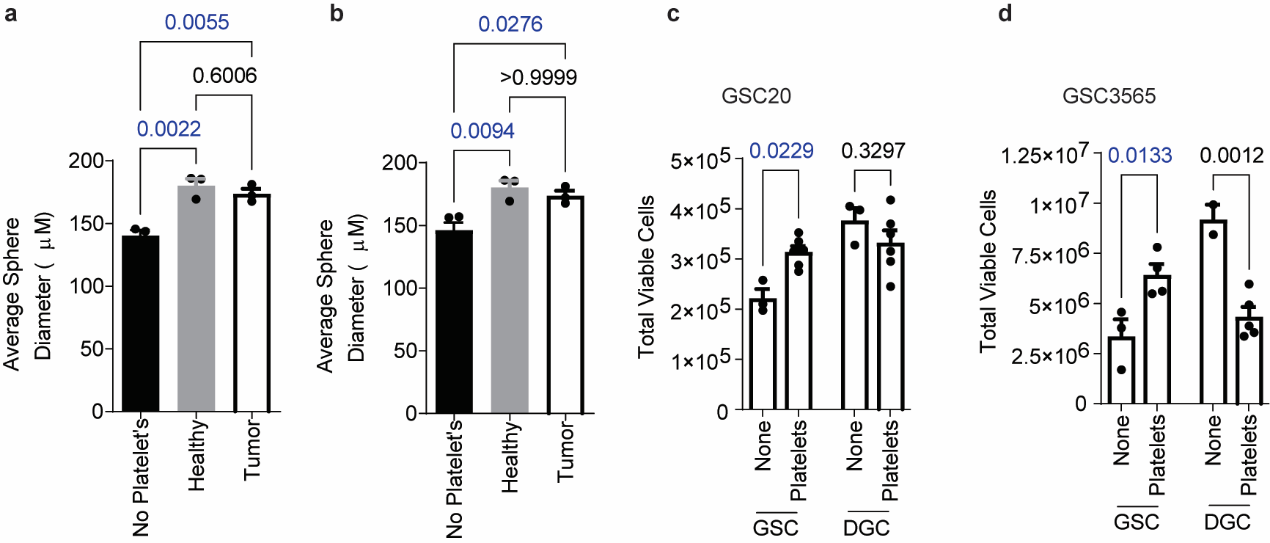


**Supplementary Fig. 3: Platelets specifically enhance GSC growth.**


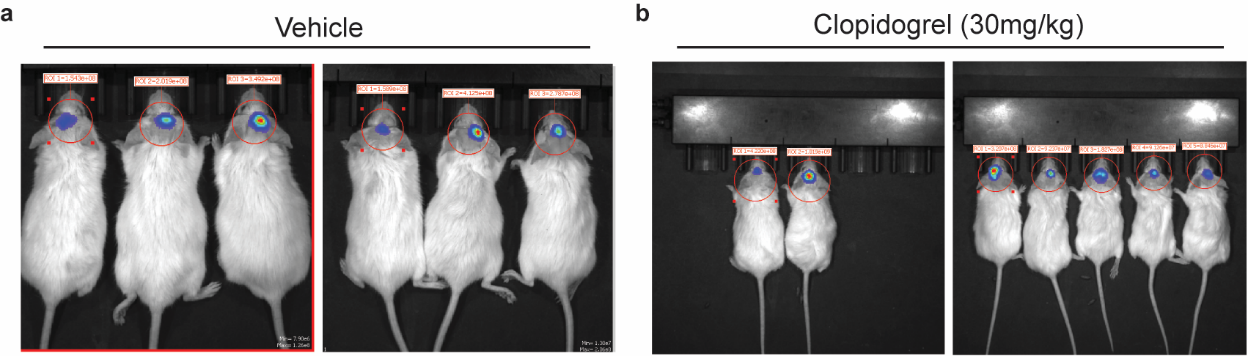


**Supplementary Fig. 4: U87 intracranial xenograft tumor formation.**


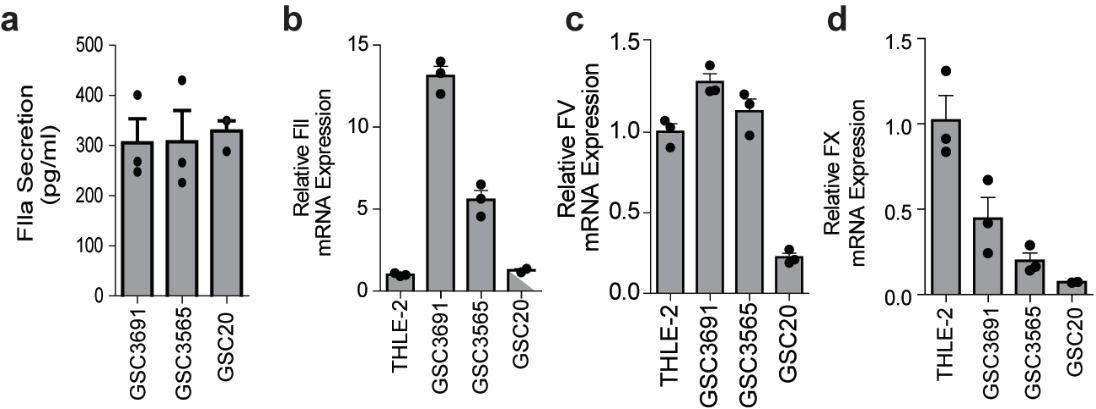


**Supplementary Fig. 5: Prothrombinase coagulation factor mRNA expression.**

**
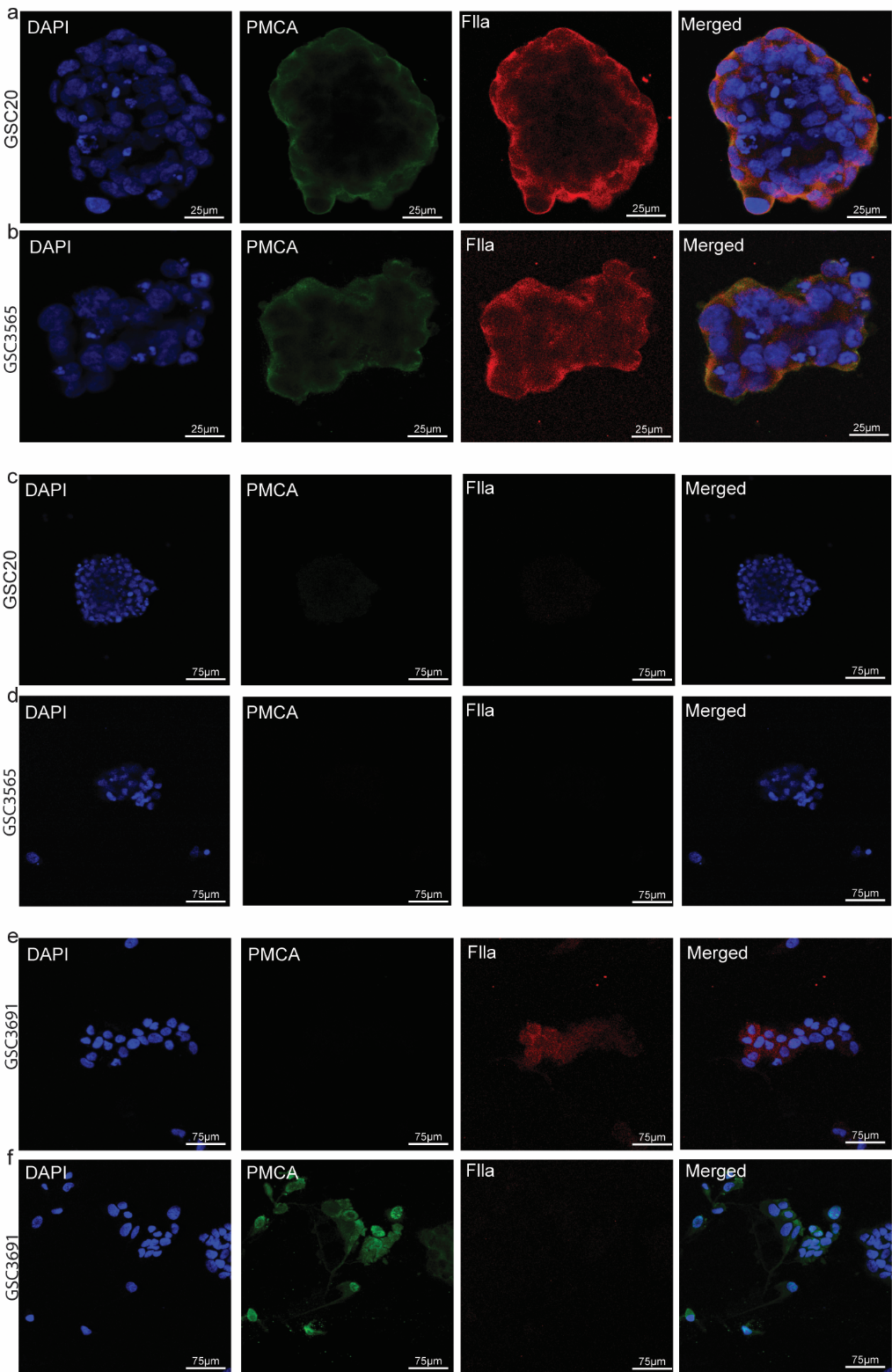
**

**Supplementary Fig. 6: Expression of thrombin in GSCs.**

**a**

**
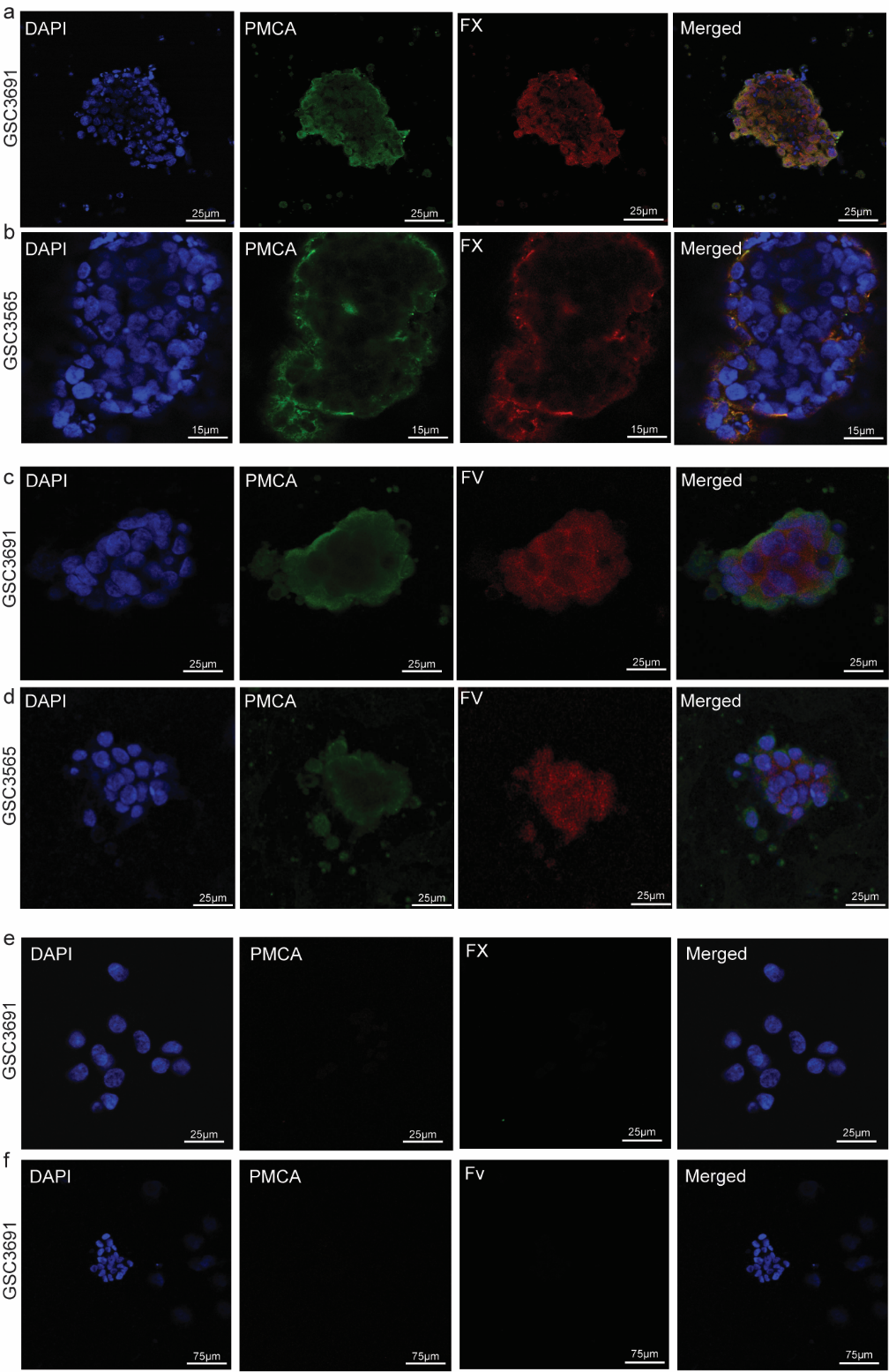
**

**Supplementary Fig. 7: Expression of FX and FV in GSCs.**

**
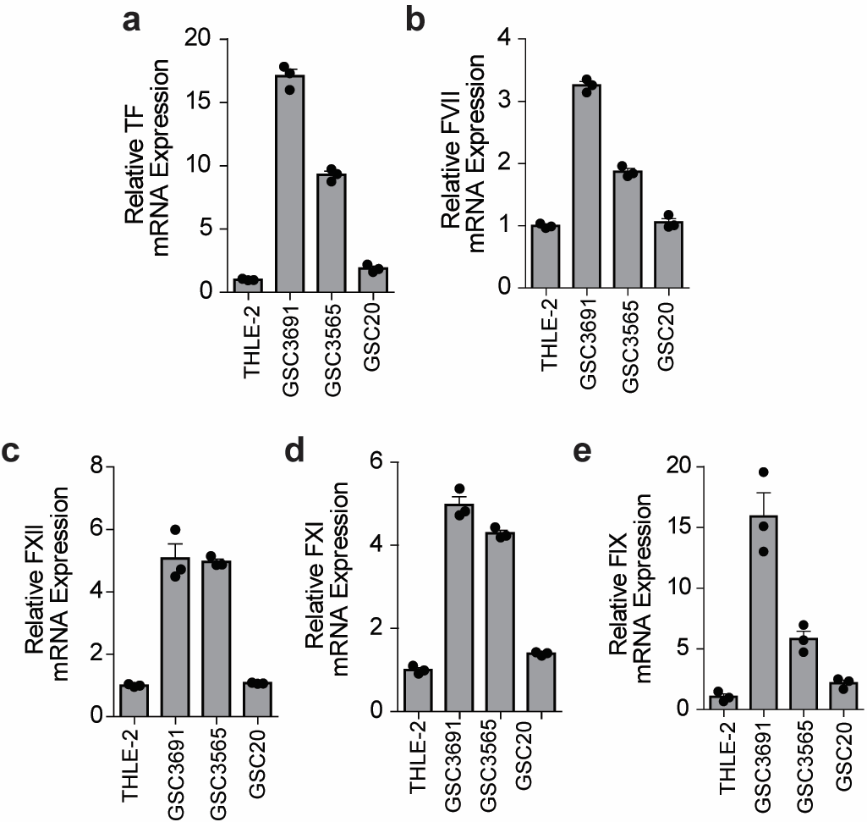
**

**Supplementary Fig. 8: Coagulation factor mRNA expression of the intrinsic and extrinsic cascade.**


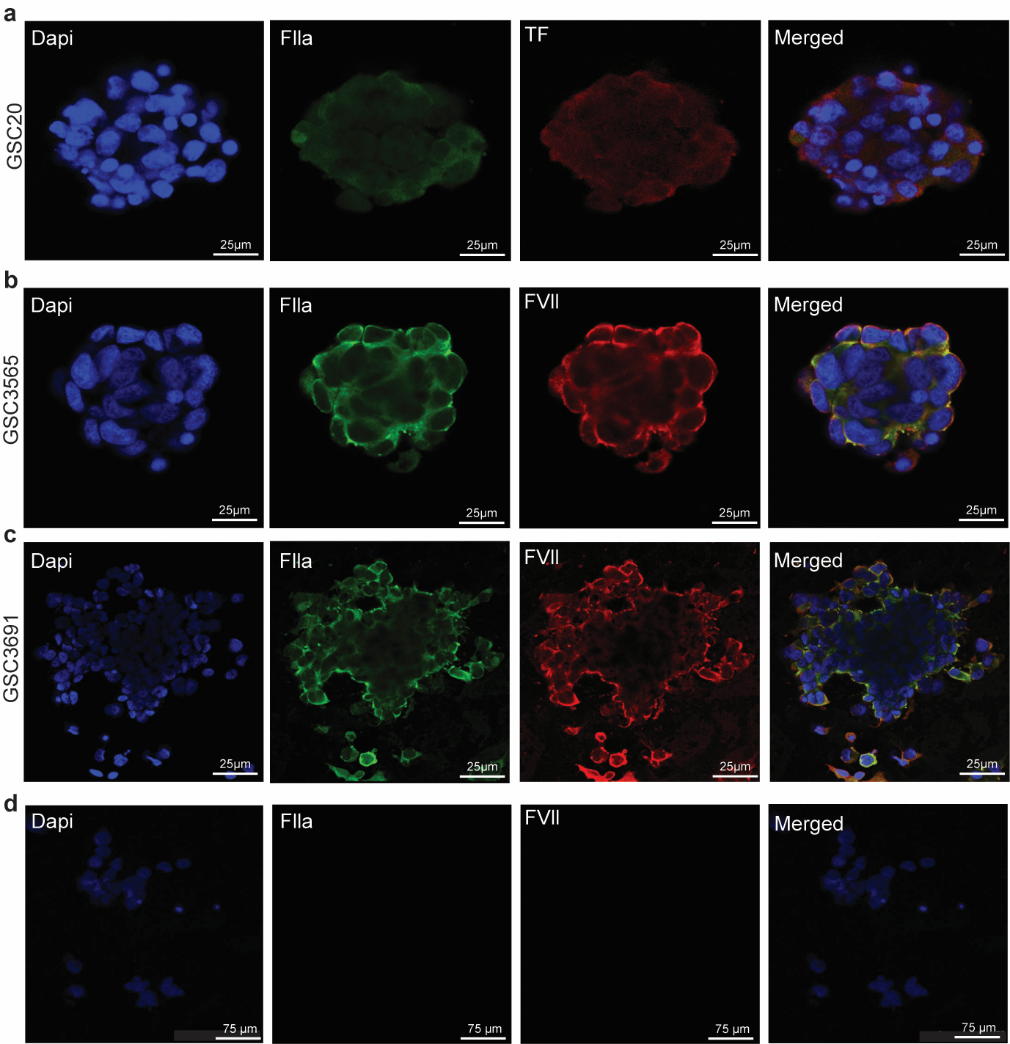


**Supplementary Fig. 9: Expression of factors of the extrinsic coagulation pathway in GSCs.**

**
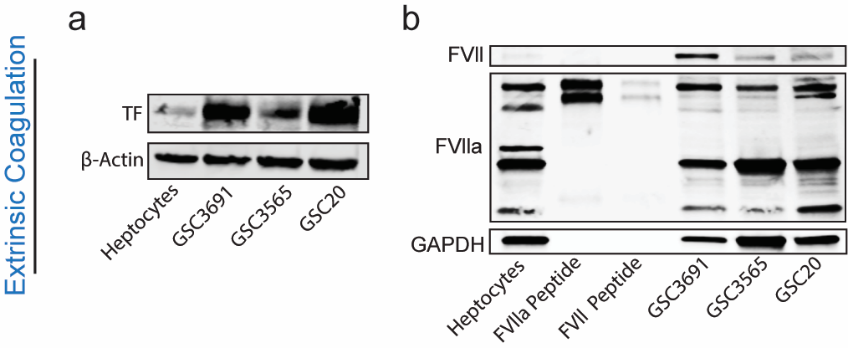
**

**Supplementary Fig. 10: Expression of factors of the extrinsic coagulation pathway.**

**
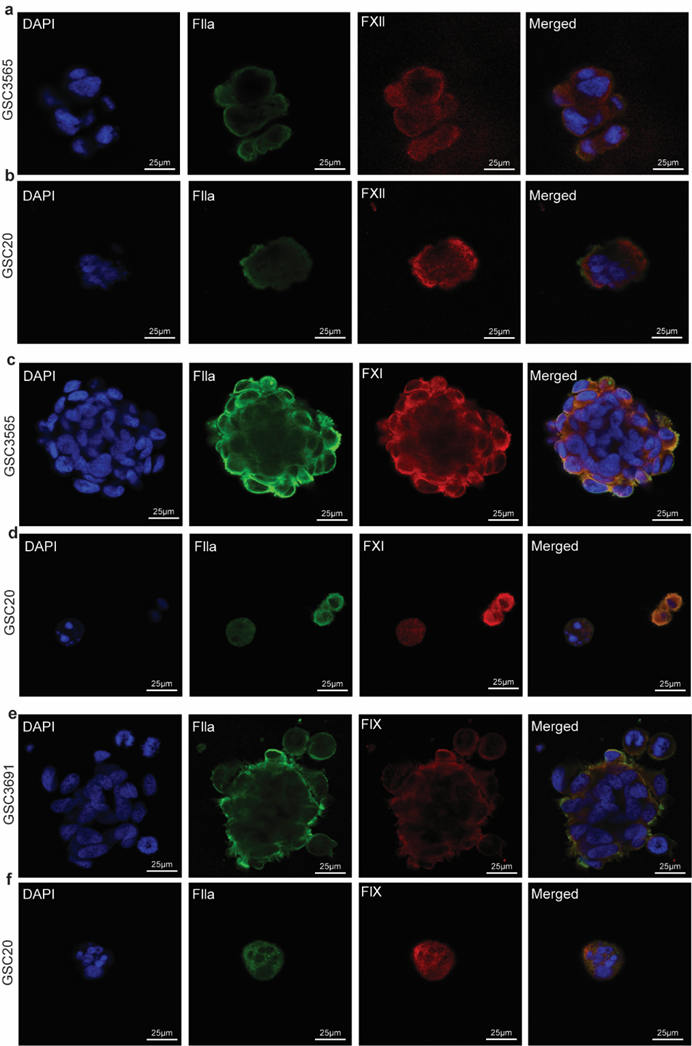
**

**Supplementary Fig. 11: Expression of factors of the intrinsic coagulation pathway in GSCs.**


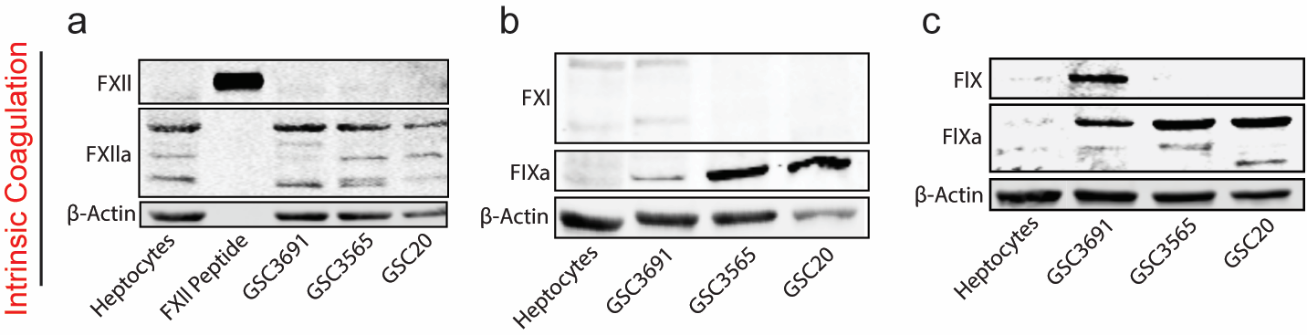


**Supplementary Fig. 12: Expression of factors of the intrinsic coagulation pathway.**


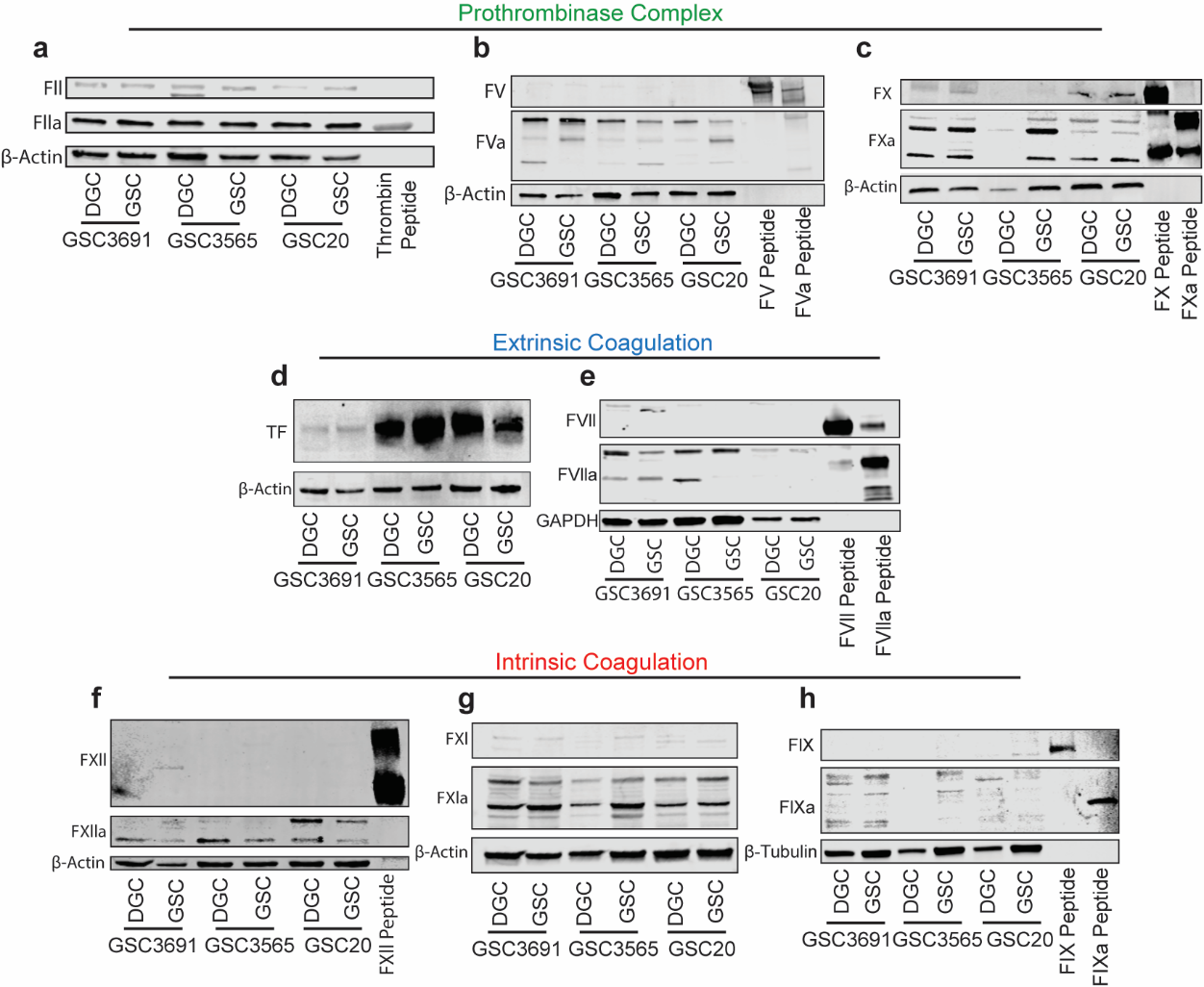


**Supplementary Fig. 13: Expression of the coagulation cascade in GSCs and DGCs.**


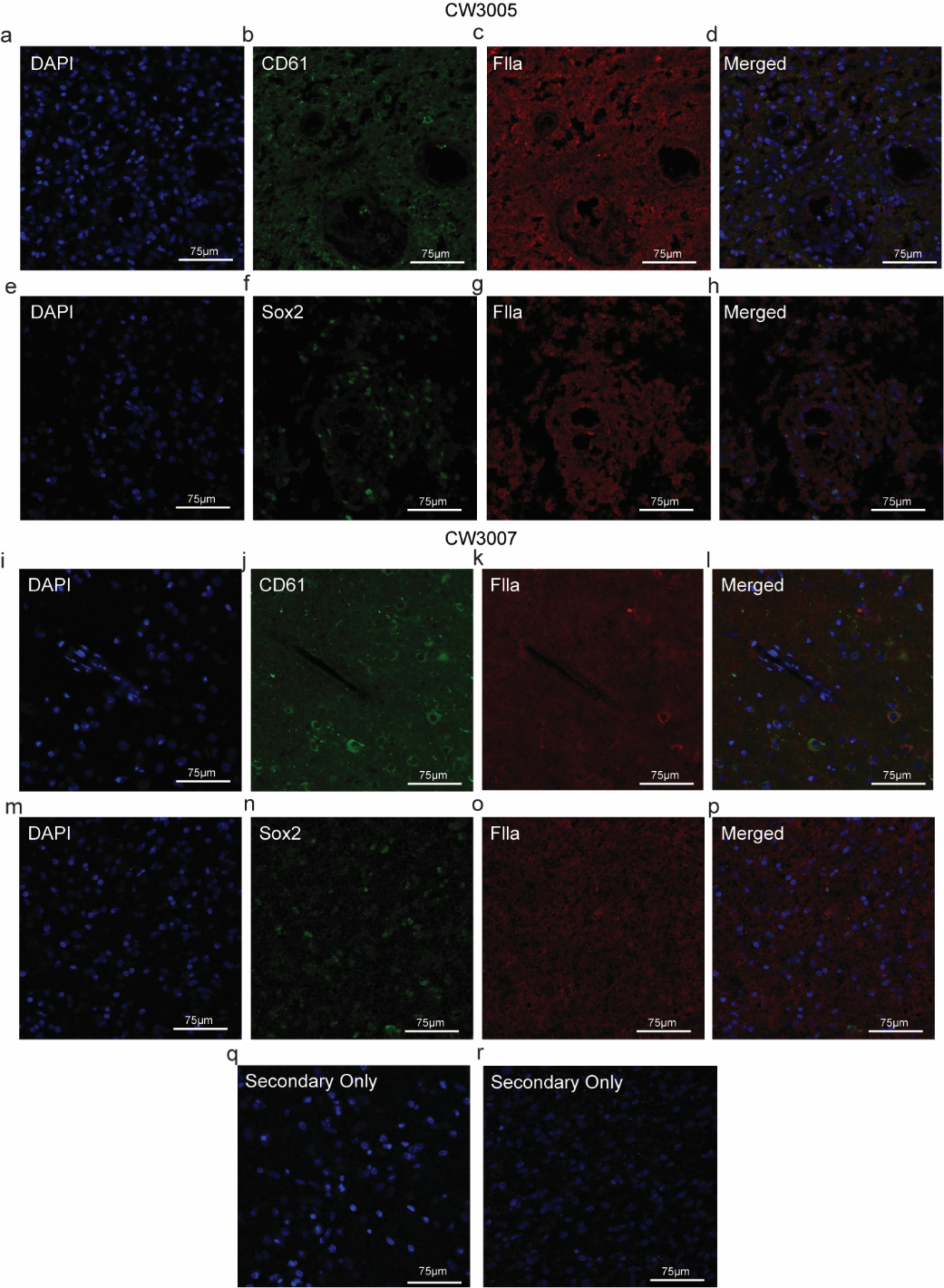


**Supplementary Fig. 14: Expression of thrombin in GSC and platelet containing niches in GBM patient tissue.**

|  | **Platelet Count (per microliter of blood)** | |  |
| --- | --- | --- | --- |
|  | High (350+) | Normal (150-300) | *p*-value |
|  | | | |
| n | 20 | 204 |  |
|  |  |  |  |
| Age at diagnosis, mean (SD) | 66.95 (9.09) | 62.00 (1.75) | 0.069 |
|  |  |  |  |
| Sex, N (%) |  |  |  |
| Male | 14 (70.0%) | 69 (34.2%) | 0.003 |
| Female | 6 (30.0%) | 133 (65.8%) |  |
|  |  |  |  |
| Race (%) |  |  |  |
| Asian/Other | 1 (5.0%) | 2 (1.0%) | 0.295 |
| Black | 1 (5.0%) | 10 (4.9%) |  |
| Asian | 18 (90.0%) | 192 (94.1%) |  |
|  |  |  |  |
| EOR, n (%) |  |  |  |
| Biopsy | 2 (10.0%) | 14 (6.9%) | 0.656 |
| Gross total | 12 (60.0%) | 112 (52.0%) |  |
| Subtotal | 6 (30.0%) | 78 (26.0%) |  |
|  |  |  |  |
| KPS, n (%) |  |  |  |
| <70 | 6 (30.0%) | 45 (22.1%) | 0.397 |
| >=70 | 9 (45.0%) | 106 (52.0%) |  |
| Unknown | 5 (25.0%) | 53 (26.0%) |  |
|  |  |  |  |
| Recurrence, n (%) |  |  |  |
| NO | 11 (55.0%) | 81 (39.7%) | 0.235 |
| Yes | 9 (45.0%) | 123 (60.3%) |  |
|  |  |  |  |
| Vital Status, n (%) |  |  |  |
| Alive | 1 (5.0%) | 18 (8.8%) | 0.999 |
| Dead | 19 (95.0%) | 186 (91.2%) |  |

**Supplementary Table 1. Descriptive statistics stratified by platelet count category**

|  | Univariate HR (95% CI) | *p*-value | Multivariable* OR (95% CI) | *p*-value |
| --- | --- | --- | --- | --- |
| **Overall Survival** |  |  |  |  |
| High Platelet (350+) | 1.81 (1.12-2.91) | 0.015 | 1.45 (0.89-2.35) | 0.134 |
| Normal Platelets (150-350) | Ref |  | Ref |  |
| ***Adjusted for age at Diagnosis** | | | | |

**Supplementary Table 2: Cox proportional hazard results: Overall survival & time to recurrence**

|  | Median Survival Time (Months) (95% CI) | *p – value* |
| --- | --- | --- |
| **Platelet Category** |  |  |
| High Platelet (350+) | 6.85 (4.03-19.70) | 0.014 |
| Normal Platelets (150-350) | 12.33 (11.02-13.80) |  |

**Supplementary Table 3: Median survival by platelet category**

| **Quantitative PCR Primers** | |
| --- | --- |
| **TF:** | |
| Forward | 5’-CAGAGTTCACACCTTACCTGGAG-3’ |
| Reverse | 5’-GTTGTTCCTTCTGACTAAAGTCCG-3’ |
| **FV:** | |
| Forward | 5’-GCCAGACCTTGCTGGAAAATGG-3’ |
| Reverse | 5’-CCAACCTCTGTGTTTAGGAGCC-3’ |
| **FX:** | |
| Forward | 5’-TGGTGGAACCATTCTGAGCGAG-3’ |
| Reverse | 5’-CGGTTGTGCTTGATGACCACCT-3’ |
| **FXll:** | |
| Forward | 5’-CTCTGTCCACAACACCTCACTG-3’ |
| Reverse | 5’-ATCAGGACCCTTGCACTGGCAT-3’ |
| **FXl:** | |
| Forward | 5’-GTGACCAACGAAGAGTGCCAGA-3’ |
| Reverse | 5’-CCAGACCTCATTGTGTTTGCAGG-3’ |
| **FlX:** | |
| Forward | 5’-GGTGGAGAAGATGCCAAACCAG-3’ |
| Reverse | 5’-CAACACAGTGGGCAGCAGTTAC-3’ |
| **Fll:** | |
| Forward | 5’-GGTGCGCATTGCCAAGCA-3’ |
| Reverse | 5’-AGGGTCCCCCACTGTCAC-3’ |
| **FVll:** | |
| Forward | 5’-CCTCAAGTCCATGCCAGAATG-3’ |
| Reverse | 5’-CACAGATCAGCTGGTCATCCT-3’ |
| **Oct4:** | |
| Forward | 5’-TCTCCCATGCATTCAAACTGA-3’ |
| Reverse | 5’-CCTTTGTGTTCCCAATTCCTTC-3’ |
| **NANOG:** | |
| Forward | 5’-GAAATACCTCAGCCTCCAGC-3’ |
| Reverse | 5’-GCGTCACACCATTGCTATTC-3’ |
| **CD61:** | |
| Forward | 5’-CACTGAGAGCAGGACCACCAG-3’ |
| Reverse | 5’-CTAGTGGAAAGTCCATCCTGTATGTG-3’ |
| **Actin:** | |
| Forward | 5’-AGAAAATCTGGCACCACACC-3’ |
| Reverse | 5’-AGAGGCGTACAGGGATAGCA-3’ |

**Supplementary Table 4: qPCR forward and reverse primers**
