# Supplementary material for: Glioma stem cells activate platelets by plasma-independent thrombin production to promote glioblastoma tumorigenesis
Source: Neurooncol Adv. 2022 Nov 7;4(1):vdac172. doi: 10.1093/noajnl/vdac172 (PMC9700385; doi:10.1093/noajnl/vdac172)
Supplement: vdac172_suppl_Supplementary_Data_S2 [file vdac172_suppl_supplementary_data_s2.docx]

**Supplementary Material and Methods**

**Patient Samples**

All human patient tumor tissue and blood samples were acquired from newly diagnosed or recurrent GBM patients 18 and older seen for clinical care at University Hospitals-Seidman Cancer Center in compliance with protocols approved by the University Hospitals Institutional Review Board (IRB) and proper informed consent. All sample information such as patient demographics (gender, age, tumor stage, tumor grade and prior treatments) were obtained as de-identified information associated with the study number for each sample. Tissue from non-lesional epilepsy specimens was similarly acquired in accordance with IRB approved protocols. Blood from healthy control subjects was provided following University Hospital IRB guidelines and was collected at University Hospitals Cleveland Medical Center. Tumor and epilepsy tissues were flash frozen at the time of collection for either western blot analysis or DNA isolation and subsequent PCR assays. Blood was processed for platelet isolation immediately after collection.

**Patient-derived glioma stem cell lines**

The GSC3565 and GSC3691 cell lines were generous gifts from Dr. Jeremy Rich (UPMC) and the GSC20 cell line was a generous gift from Dr. Erik Sulman (NYU). These stem cell lines were derived from freshly resected GBM specimens, established, and maintained as previously published ^1,2^. Regarding the transcriptional subtype classification of the cells, GSC3565 and GSC3691 cells are proneural, whereas GSC20 cells are mesenchymal. Briefly, cells were grown in suspension flasks (CytoOne, CC-672-4175, USA Scientific, Ocala, FL) in serum-free neuro medium (MACs neuro medium with Neurobrew-21 (Miltenyi Biotec Inc., 130-093-570 and 130-097-263, respectively, Auburn, CA), 20 ng/mL EGF (PeproTech, AF-100-15, Rocky Hill, NJ) and 20 ng/mL FGF (PeproTech, 100-18B, Rocky Hill, NJ)) supplemented with 1% penicillin-streptomycin (ThermoFisher Scientific, 15140122, Waltham, MA) and 1% L-glutamine (ThermoFisher Scientific, 25030081,Waltham, MA) at 37°C in a humidified incubator with 5% CO_2_. (ATCC, 30-1012K, Cheyenne, WY). STR validation was consistently conducted on all of our cultures.

To generate DGCs, GSCs were differentiated in serum-free neural medium, as described above, supplemented with 10% FBS to promote GSC differentiation into DGCs. StemPro^®^ Neural Stem Cells (Gibco, cat. no. A10509-01) were obtained, grown in NSC culture medium, and used in the proliferation assays. Normal human astrocytes (ScienceCell, 1800, Carlsbad, CA) were obtained and grown in astrocyte medium (ScienceCell, 1801, Carlsbad, CA), and used in the proliferation assays.

**Non-Neoplastic Cells:**

Astrocytes (Normal human astrocytes, ScienceCell, Cat. #: 1800) were acquired from Science Cell and grown according to the ScienceCell protocols. Neural stem cell line (StemPro Neural Stem Cells, GIBCO, Cat. #: A15654) were acquired from GIBCO and grown according to the Gibco protocol. THLE 2, human liver epithelial cells (ATCC, Cat #: CRL-2706) were acquired from ATCC and grown according to the ATCC protocol.

**Peripheral Blood Collection/Platelet Activation**

Blood was drawn from consented patients and normal controls using a 23-gauge butterfly cannula; the first 1 mL was discarded before drawing an additional 10 ml of blood into a polypropylene vial containing 3.2% sodium citrate (final dilution 1:9). Blood was transferred to a sterile 15 ml conical tube (Fisher Scientific, 339651, Hampton, NH). Blood was centrifuged at 200 ×*g* for 20 minutes at room temperature, and platelet-rich plasma (PRP) was transferred to a sterile 15 ml conical tube. Prostaglandin E2 (Millipore Sigma, P5640, Burlington, MA) was added to the PRP at a concentration of 1 µg/mL to temporarily prevent platelet activation; the treated PRP was then centrifuged at 100 x*g* for 20 minutes at room temperature to remove contaminate cells. The PRP was decanted from the pellet of contaminant cells into a new sterile 15 ml conical tube and centrifuged again at 1400 x*g* for 5 minutes at room temperature to pellet the platelets. Platelet pellets were washed with 1X Hanks Buffered Salt Solution (HBSS, ThermoFisher, 14175-095, Waltham, MA) and centrifuged at 1400 x*g* for 5 minutes at room temperature. The platelet pellets were gently resuspended in Tyrode’s buffer (Boston BioProducts, C-4110, Ashland, MA) and platelet count was determined using the Beckman Z2 particle counter (Beckman Coulter Inc., Indianapolis, IN) before immediate use in assays.

**Immunofluorescence Staining**

GBM tissues were fixed in 4% paraformaldehyde overnight at 4°C, followed by overnight incubations in graded sucrose (15% and 30%) prior to embedding in OCT compound (Fisher Scientific, 4585, Hampton, NH) and subsequent freezing on dry ice. Frozen samples were sectioned at 20 µm with the Leica 1850 Cryostat (Leica, CM1850, Buffalo Grove, IL) and mounted on *Superfrost® Plus* slides (Fisher Scientific, 12-550-15, Hampton, NH). Sections were rinsed with phosphate-buffered saline (PBS, pH 7.4), followed by incubation in 10% normal goat serum in PBS (NGS, Millipore Sigma, G6767, St. Louis, MO) for 30 minutes at room temperature to block nonspecific binding sites prior to the application of primary antibodies. Primary Antibodies are included in supplemental methods. The primary antibodies used were specific for thrombin (1:100), PMCA (1:50), TF (1:100), and FVIIa (1:75) (Abcam; ab20877, ab3528, ab228968, and ab61412, respectively, Cambridge, MA); FX/FXa (1:75) and FV (1:75) (ThermoFisher Scientific; PA5-22059 and PA5-81998, respectively, Waltham, MA); FXl (1:75) (Bioss Antibodies, bs-10336R, Woburn, MA); and CD61 (1:75) (Novus Biologicals, HLI1203, Littleton, CO). Primary antibodies were incubated overnight at 4◦C followed by incubation in the appropriate Alexa Fluor secondary (1:300) for one hour at room temperature (Alexa Fluor -488 A32723 mouse; A11008 rabbit; Alexa Fluor – 594 A11005 mouse; A11037 rabbit; Alexa Fluor- 790 A11357 mouse, ThermoFisher Scientific, Waltham, MA). Slides were mounted with DAPI fluoromount-G*®* (SouthernBiotech, 0100-20, Birmingham, AL) and visualized on a Leica TCS SP8 gats STED 3X confocal microscope.

**TCGA bioinformatics analysis**

Raw RNA-Seq counts were sourced from the TCGA-GBM dataset into the R programming language via the TCGAbiolinks library ^3^. Count values were then TMM normalized and scaled to counts per million values by the edgeR library ^4^. Pathway enrichment values were generated by the GSVA library using the Poisson kernel and the Molecular Signatures Database chemical and genetic perturbations (CGP) set ^5,6^. Enrichment values were scaled by a factor of 10 to aid in interpreting the hazard ratio intervals. The pat­hways ‘HARRIS_HYPOXIA’ and ‘RAGHAVACHARI_PLATELET_SPECIFIC_GENES’ were selected to represent hypoxia and platelet signatures, respectively, from the set ^7-9^. Progression-free intervals were retrieved from the TCGA-CDR with Cox proportional hazards modeling used to determine the impact platelet marker gene expression on patient survival ^10^. Enrichment scores for survival analysis were stratified into quantiles to the continuous range into the bottom third (-0.587, -0.208, referred to as low expression in text), middle third (-0.208, 0.216 referred to as normal expression in text), and upper third (0.216, 0.587, referred to as high expression in text).

**GBM-Patient survival analysis**

Thrombocyte counts were categorized as high (greater than 350 per 100,000 cells) and normal (150-300 per 100,000 cells) among GBM cases. Demographic and clinical characteristics were evaluated between thrombocyte levels. T tests and chi squared tests were performed to assess differences in continuous and categorical variables, respectively. Kaplan Meier analyses were performed and median survival in months, with corresponding 95% confidence intervals, are reported. Univariate and multivariable Cox proportional hazards were performed to assess the impact of thrombocyte count on overall survival, and hazard ratios are reported. Predictors were not found in violation of the proportional hazards assumption. Multivariable model was adjusted for age at diagnosis. All survival analyses were performed in R version 3.5.0. P values less than 0.05 are considered statistically significant.

**Western blot analysis**

Flash-frozen tissue samples or cell line pellets were homogenized in 1X lysis buffer (Cell Signaling Technology, 9803, Danvers, MA) containing halt protease and phosphatase inhibitors (Thermo Scientific, 78443, Waltham, MA) on ice for 10 minutes prior to centrifuging at 1400 xg at 4◦C for 10 minutes. Supernatant was collected and the protein concentration was determined with the Pierce BCA protein assay kit (Thermo Scientific, 23227, Waltham, MA) following the manufacturer's protocol. Thirty micrograms of protein per sample was loaded in Laemmli sample buffer containing 2-Mercaptoethanol onto a precast Mini-Protean TGX gel, 4-10% (Bio-Rad, 456-1094, Hercules, CA) and separated at 100 volts before transfer to a PDVF membrane (Immobilon-FL, Millipore Sigma, IPFL00010, St. Louis, MO) via the wet transfer method at 80 volts for two hours. Antibodies used are in supplementary methods. The membrane was subsequently rinsed in Tris-buffered saline (TBS; 50 mM Tris-HCl 150 mM NaCl, pH 7.6) and blocked in Intercept blocking buffer for one hour (Li-Cor, 927-50000, Lincoln, NE) prior to the application of primary antibodies targeting CD61 (1:1000) (Novus Biologicals, NBP2-67416, Littleton, CO); FVlla (1:1000), FV (1:1000), FXll(1:750), FlX(1:1000), and TF(1:1000) (Abcam; ab61412, ab108614, ab242123, ab255824, and ab228968, respectively, Cambridge, MA); FX/FXa (1:1000) (ThermoFisher, PA5-22059, Waltham, MA); Thrombin/Prothrombin (1:750) (Haematologic Technologies, X0505, Essex Junction, VT). GAPDH (1:1000) (Santa Cruz Biotech Inc., SC47724, Dallas, TX); and Actin (1:1000) (Millipore Sigma, MAB1501, St. Louis, MO) were used as loading controls. The membrane was incubated overnight at 4°C with the primary antibody, rinsed with TBS-T (TBS; 50 mM Tris-HCl 150 mM NaCl, 1 % Tween-20 v/v% pH 7.6) followed by incubation in secondary (IRDye 680- and IRDye 800CW-conjugated secondary antibodies, Li-Cor, 926-68072 and 926-32213, respectively, Lincoln, NE) for one hour at room temperature prior to visualization on the Li-Cor Odyssey CLx imaging System (Li-Cor, Lincoln, NE). Western blot analysis of band intensity was completed using Image Studio Analysis software from Li-Cor.

**Quantitative real time PCR (qPCR) to measure stem cell marker expression in GSCs upon platelet exposure**

500,000 GSCs were treated with platelets at a 1:10 GSC:platelet ratio for three days at 37°C in a humidified incubator with 5% CO_2._ GSCs were then rinsed with HBSS. RNA was extracted from GSCs using an RNeasy Mini kit (Qiagen, 56605147,Germantown, MD ), GSC RNA quantity and quality was assed using a NANODROP Lite spectrophotometer (Thermofisher, ND-Lite, Waltham, MA) and reverse transcribed using a cDNA synthesis kit (BIO-RAD, 1708891, Hercules, CA). Assay plating and RNA extraction methodology is included in supplementary methods. To determine the expression of GSC markers, platelet markers, and coagulation factors, qPCR was run using the SYBR Green Supermix protocol (BIO-RAD, 1725270, Hercules, CA) and BIO-RAD CFX Connect Real Time System. Amplification was performed with an initial denaturing step at 96◦C for 5 minutes followed by denaturing at 94◦C for 30 seconds and annealing for 30 seconds using SsoAdvanced SYBR Green (Bio-Rad Laboratories, Hercules, CA #1725270). 20 μl samples were loaded in triplicate and run for 40 cycles. Quantification was done using the ΔΔC_T_ method. Please refer to Supplementary Table 4 for PCR primer sequences and annealing temperatures.

**Human thrombin ELISA**

GSC thrombin secretion was measured using a human thrombin simplestep ELISA^®^ Kit and the subsequent protocol (ABCAM, ab270210, Cambridge, MA). In short, 1 million GSCs were plated into T725 Flask with 10ml of serum free neural medium (described above). Following 7 days, the conditioned medium was collected by centrifuging the medium cell mixture at 1300g for 4 minutes; conditioned medium was removed and saved for ELISA. 50µl of conditioned medium was used per well for the thrombin ELISA analysis along with 50µl of the antibody cocktail. Following 1 hr. incubation, the wells were washed with 1X wash buffer, which came with the kit, and 100µl of TMB development solution was added to each well followed by 100µl of stop solution. The OD absorbance was recorded at 450nm using a Synergy HT plate reader. Background absorbance signals from samples containing only medium were subtracted to determine GSC thrombin secretion.

**Functional assays to measure GSC function upon platelet exposure**

***Proliferation assay:*** Proliferation assays using the CellTiter Glo^®^ Luminescent Cell Viability Assay were conducted (Promega, G7571, Madison, WI). This assay produces a luminescent signal that is proportional to the amount of ATP present and correlates to the number of cells present. Briefly, one thousand GSCs, DGCs or Neural Stem cells were incubated with platelets from either healthy subjects or GBM patients at a GSC:platelet ratio of 1:10, 1:20, 1:50, or 1:100, at 37°C in a humidified incubator with 5% CO_2_ for either 1,3,5 or 7 days. CellTiter Glo^®^ was added to each treatment parameter, to lyse the culture, and incubated for 10 minutes at room temperature prior to reading the luminescence on a Synergy HT plate reader (BioTek, Winooski, VT), directly measuring the amount of ATP present in the culture. Background luminescence signals from samples containing only platelets were subtracted to determine GSC growth.

***Colony formation assay:*** GSC lines were incubated with platelets from either healthy subjects or tumor patients for a colony formation assay. Briefly, 200,000 cells were treated with platelets at a 1:10 GSC:platelet ratio for three days at 37°C in a humidified incubator with 5% CO_2._ GSCs were then rinsed with HBSS, resuspended in methylcellulose media and 12,000 cells/well were plated into a 6 well plate (Fisher Scientific, 09-201-598, Hampton, NH). Plates were then incubated at 37°C in a humidified incubator with 5% CO_2_ for 10- 14 days prior to measuring the sphere diameter using the Gel count System (Oxford Optronix, London, ON) as previously published ^39^.

**Platelet aggregation assay**

PRP (450 µL) as previously described was loaded into a microcuvette and incubated for 5 minutes at 37°C. Platelet aggregation was initiated by adding 50 µL of 2 units/ml human thrombin (Haematologic Technologies, JJ0701, Essex Junction, VT) or conditioned media and monitored for 30 minutes in a two-channel optical aggregometer (Chrono-Log, 700-2, Havertown, PA). All platelet aggregation assays were completed within 3 hours of blood collection. Data analysis consisted of paired comparisons of the levels of platelet aggregation between untreated and thrombin treated platelets.

**Flow cytometry assay to measure platelet activation**

Platelets were incubated in various doses of GSC conditioned medium (10%, 50%, 90%) for 1 hour at 37°C in a humidified incubator with 5% CO_2_. Platelet flow cytometry staining was conducted using the following labeled antibodies: anti-CD62P-PE, anti-PAC-1-FITC, and anti-CD42b-PerCP-eFluor 710 (ThermoFisher Scientific, 12-0626, 82A5-28564, 46-0429-41 respectively, Waltham, MA) added directly to the tubes with the Platelets and conditioned medium. Platelet activation was monitored using CD42b and PAC-1, which are two established platelet activation markers ^11^. PAC1 stains for the activated conformation of CD41/Cd61. Paired analysis was completed comparing the percentage of activated platelets, referring to CD42b and PAC-1 positive Platelets in each condition, to the baseline percentage of activated platelets. Baseline platelet activation was determined using unconditioned GSC medium. Flow cytometry analysis was completed using FLOWJO v10 (FlowJo, v10, Ashland, OR).

**Low molecular weight heparin treatment**

GSC were plated at a density of 1 million GSCs in 10mls of neural medium, as described above and treated with low molecular weight heparin (Sigma, H-3149, St. Louis, MO) daily at the following doses: 100ng, 250ng, 500ng, 1000ng, 10µg, 100µg, and 500µg. GSCs were treated with their respective dose for 6 consecutive days; on the 7^th^ day GSCs were collected and protein lysates were prepared as described above. Western blot analysis was conducted for thrombin and FX (as described above) followed by flow cytometry for platelet activation (as described above).

***In vivo* Flank Antithrombin Experiments**

GSC3691 (1.0x10^6^) cells were suspended in HBSS and injected at a ratio of 1:1 with geltrex (GIBCO, A14133-02, Amarillo, TX) for a total of 200ul in the subcutaneous space along the animals right flank. Over the next 6-7 days tumors developed to the point where a small mass was visual. A digital caliper measurement (length and width) were taken every other day for a period of 11-14 days and tumor volume was calculated and normalized to day 1 of treatment. Animals received daily IP injections of either 10 mg/kg apixaban, 30 mg/kg dabigatran, or vehicle control. The experiment was terminated at the 11-day mark; tumors were harvested followed by protein lysate preparation and thrombin anti thrombin ELISA (ABCAM, ab108907, Waltham, MA). Ellipsoid tumor volume was determined using the equation: Ellipsoid Volume = 4/3π (w/2)^2^ (h/2)^2^. All animal experiments were in accordance with Case Western Reserve University IACUC policies.

**Orthotopic Xenograft model**

Intracranial xenografting was completed as previously described ^2^. U87 GBM cells were intracranial injected into 6-12 week old NSG mice. Once fully anesthetized, mice were place into a stereotaxic apparatus and incision area was prepped with betadine followed by ethanol, a small incision was made through the scalp. A 25-guage burr hole was made 2mm caudal and 3mm to the right of bregma. A 22 gauge Hamilton syringe (Fisher Scientific, 88011, Hampton, NH, USA) was inserted 3mm and retracted 0.5 mm to establish implantation pocket. Cells were injected slowly at a concentration of 5.0 x 10^4^ cell/ml for a total of 1.0x10^5^, syringe was help in place for 3-4 minutes and incision was closed with surgical glue and non-dissolvable sutures. Following 4-6 days post implantation, bioluminescent imaging was conducted to confirm tumor formation and treatments started. Mice were treated for 21 days with IP injections of either 30mg/kg clopidogrel, 10 mg/kg apixaban, 30 mg/kg dabigatran, or vehicle control. All animal experiments were in accordance with Case Western Reserve University IACUC policies. Survival analysis was completed using PRISM graphpad 9.1.2 and comparison were made using Log-Rank (Mantel-Cox) test.

**Statistical analysis**

All grouped data throughout this manuscript are presented as the mean ± standard error of the mean. All experiments reported are from independent experiments (n=3). Intergroup differences were analyzed by two-way Student’s *t*-test, when comparing two groups or one-way ANOVA with Bonferroni’s post hoc analysis, when comparing three or more groups, using GraphPad Prism software. Kaplan-Meier survival analysis was conducted using GraphPad Prism software. Significance was defined as having a p-value of *p*<0.05.

**References:**

**1.** Seidel S, Garvalov BK, Wirta V, et al. A hypoxic niche regulates glioblastoma stem cells through hypoxia inducible factor 2 alpha. *Brain.* 2010; 133(Pt 4):983-995.

**2.** Kerstetter-Fogle AE, Harris PLR, Brady-Kalnay SM, Sloan AE. Generation of Glioblastoma Patient-Derived Intracranial Xenografts for Preclinical Studies. *Int J Mol Sci.* 2020; 21(14).

**3.** Colaprico A, Silva TC, Olsen C, et al. TCGAbiolinks: an R/Bioconductor package for integrative analysis of TCGA data. *Nucleic Acids Res.* 2016; 44(8):e71.

**4.** Robinson MD, McCarthy DJ, Smyth GK. edgeR: a Bioconductor package for differential expression analysis of digital gene expression data. *Bioinformatics.* 2010; 26(1):139-140.

**5.** Liberzon A, Subramanian A, Pinchback R, Thorvaldsdóttir H, Tamayo P, Mesirov JP. Molecular signatures database (MSigDB) 3.0. *Bioinformatics.* 2011; 27(12):1739-1740.

**6.** Liu J, Lichtenberg T, Hoadley KA, et al. An Integrated TCGA Pan-Cancer Clinical Data Resource to Drive High-Quality Survival Outcome Analytics. *Cell.* 2018; 173(2):400-416.e411.

**7.** Harris BH, Barberis A, West CM, Buffa FM. Gene Expression Signatures as Biomarkers of Tumour Hypoxia. *Clin Oncol (R Coll Radiol).* 2015; 27(10):547-560.

**8.** Raghavan S, Snyder CS, Wang A, et al. Carcinoma-Associated Mesenchymal Stem Cells Promote Chemoresistance in Ovarian Cancer Stem Cells via PDGF Signaling. *Cancers (Basel).* 2020; 12(8).

**9.** Raghavachari N, Xu X, Harris A, et al. Amplified expression profiling of platelet transcriptome reveals changes in arginine metabolic pathways in patients with sickle cell disease. *Circulation.* 2007; 115(12):1551-1562.

**10.** Navred K, Martin M, Ekdahl L, et al. A simplified flow cytometric method for detection of inherited platelet disorders-A comparison to the gold standard light transmission aggregometry. *PLoS One.* 2019; 14(1):e0211130.

**11.** Yun SH, Sim EH, Goh RY, Park JI, Han JY. Platelet Activation: The Mechanisms and Potential Biomarkers. *Biomed Res Int.* 2016; 2016:9060143.
