# Supplementary material for: Glioma stem cells activate platelets by plasma-independent thrombin production to promote glioblastoma tumorigenesis
Source: Neurooncol Adv. 2022 Nov 7;4(1):vdac172. doi: 10.1093/noajnl/vdac172 (PMC9700385; doi:10.1093/noajnl/vdac172)
Supplement: vdac172_suppl_Supplementary_Data_S3 [file vdac172_suppl_supplementary_data_s3.docx]

**Supplementary figure legends:**

**Supplementary Fig. 1: Platelet and GSC expression in GBM patient tissue.** Localization of platelets and GSCs in GBM patient tissues (cases CW3007 and CW2987). Immunofluorescence (IF) staining of (**a, f**) nuclei, (**b, g**) GSCs, (**c, h**) endothelial cells/platelets, and (**d, i**) platelets only. (**e, j**) Merged image demonstrating GSCs encompassed by platelets in the perivascular and nonperivascular regions of GBM patient tissues. (**k, l, m**) IF staining negative control (secondary antibody only), for each of the patient samples used in this analysis. (Scale = 10 µm)

**Supplementary Fig. 2: CD61expression is specific to GBM. a,** Western blot showing that CD61 expression is higher in grade IV glioma tissue than in lower grade glioma tissue, and epilepsy control tissue. **b**, qPCR for CD61 showing that CD61 mRNA levels in GBM patient tissues positively correlated with glioma grade. **c, d,** qPCR for the mRNA levels of the GSC markers (**c**) NANOG and (**d**) OCT4, which showed direct relationships with glioma grade, in GBM patient tissues compared to control epilepsy tissue. All data are presented as the mean ± SEM from three independent experiments.

**Supplementary Fig. 3: Platelets specifically enhance GSC growth. (a, b)** Average sphere diameter of GSCs upon exposure to platelets (1 GSC : 10 platelets) from healthy subjects or GBM patients as analyzed by determining sphere diameters 10 days after platelet exposure. (**c, d**) Proliferation of GSCs and DGCs upon platelet exposure. All data are presented as the mean ± SEM from three independent experiments.

**Supplementary Fig. 4: U87 intracranial xenograft tumor formation.** (**a,b**) Sphere formation confirmation 6 days post implantation when treatments started (Vehiclel, n=6; Clopidogrel, n=7).

**Supplementary Fig. 5: Prothrombinase coagulation factor mRNA expression. a,** Flla (thrombin) ELISA of GSC-conditioned medium measuring Flla secretion by GSCs. (**b-c**) qPCR for mRNA expression of (**b**) Fll, (**c**) FV, and (**d**) FX. All data are presented as the mean ± SEM of three independent experiments. THLE-2 = human adult hepatocytes.

**Supplementary Fig. 6: Expression of thrombin in GSCs. a-h,** Expression of Flla as observed by confocal microscopy. IF staining of (**a**) Flla, PMCA, and DAPI (GSC20), (**b**) Flla, PMCA, and DAPI (GSC3565), (**c**) DAPI without primary antibodies (GSC20), (**d**) DAPI without primary antibodies (GSC3565), (**e**) Flla and DAPI only (GSC3691), and (**f**) PMCA and DAPI only (GSC3691).

**Supplementary Fig. 7: Expression of FX and FV in GSCs.** Expression of FX and FV as shown by confocal microscopy. IF staining of (**a**) FX, PMCA, and DAPI (GSC3691), (**b**) FX, PMCA, and DAPI (GSC3565), (**c**) FV, PMCA, and DAPI (GSC3691), (**d**) FV, PMCA, and DAPI (GSC3565), (**e**) GSC3691 without the anti-FX or anti-PMCA primary antibody, and (**f**) GSC3691 without the anti-FV or anti-PMCA primary antibody.

**Supplementary Fig. 8: Coagulation factor mRNA expression of the intrinsic and extrinsic cascade.** QPCR measuring mRNA expression of the coagulation factors that make up the extrinsic coagulation cascade. mRNA expression of (**a**) TF, (**b**) FVll. QPCR measuring mRNA expression of the coagulation factors that make up the intrinsic coagulation cascade. mRNA expression of (**c**) FXll, (**d**) FXl, and (**c**) FlX. All data are presented as the mean ± SEM of three independent experiments. THLE-2 = human adult hepatocytes.

**Supplementary Fig. 9: Expression of factors of the extrinsic coagulation pathway in GSCs.** Expression of TF and FVll as shown by confocal microscopy. IF staining of (**a**) TF, Flla, and DAPI (GSC20), (**b**) FVll, Flla, and DAPI (GSC3565) and (**c**) FVll, Flla, and DAPI (GSC3691), and (**d**) GSC3691 cells without anti-FVII and anti-Flla primary antibody

**Supplementary Fig. 10: Expression of factors of the extrinsic coagulation pathway.** (**a-b**) Representative western blot data for the expression of the extrinsic coagulation factors (a) Tissue Factor (TF) and (b) FVll/FVlla in GSCs.

**Supplementary Fig. 11: Expression of factors of the intrinsic coagulation pathway in GSCs:** Expression of FXll, FXl, and FlX as shown by confocal microscopy. IF staining of (**a**) FXll, Flla, and DAPI (GSC3565), (**b**) FXll, Flla, and DAPI (GSC20), (**c**) FXl, Flla, and DAPI (GSC3565), (**d**) FXl, Flla, and DAPI (GSC20), (**e**) FlX, Flla, and DAPI (GSC3565), and (**f**) FIX, Flla, and DAPI (GSC20).

**Supplementary Fig. 12: Expression of factors of the intrinsic coagulation pathway.** (**a-c**) Representative western blot data for the intrinsic coagulation factors **(a)** FXll/FXlla, **(b)** FlX/FlXa, and **(c)** FXl/FXla in GSCs.

**Supplementary Fig. 13: Expression of the coagulation cascade in GSCs and DGCs.** (**a-c**) Representative western blot data for the prothrombinase complex in GSCs and DGCs. Western blot of (**a**) Fll and Flla, (**b**) Fv and FVa, and (**c**) Fx and Fxa. (**d-e**) Representative western blot data for the extriniscs coagulation factors. Western blot for (**d**) TF and (**e**) FVll and FVlla. (**f-h**) Representative Western blot data for the intrinsic coagulation factors. Western blot of (**f**) FXll and FXlla, (**g**) FXl and FXla, and (**h**) FlX and FlXa.

**Supplementary Fig. 14: Expression of thrombin in GSC and platelet containing niches in GBM patient tissue:** Expression of (**a**) CD61, (**b**) Flla, and (**c**) Dapi in GBM patient specimens 3005. (**d**) Merged image of the staining showing expression of thrombin in areas that contain CD61 positive platelets. Expression of (**f**) Sox2, (**g**) Flla. and (**h**) Dapi in GBM patient specimens. Expression of (**i**) CD61, (**j**) Flla, and (**k**) Dapi in GBM patient specimens 3005. (**l**) Merged image of the staining showing expression of thrombin in areas that contain CD61 positive platelets. Expression of (**m**) dapi, (**n**) Sox2. and (**o**) Flla in GBM patient specimens. (**p**) Merged image of the staining showing expression of thrombin in areas that sox2 positive GSCs. (**q,r**) IF staining negative control (secondary antibody only), for each of the patient samples used in this analysis. (Scale = 75µm)
